# *Ex vivo* organotypic culture system of precision-cut slices of human pancreatic ductal adenocarcinoma

**DOI:** 10.1038/s41598-019-38603-w

**Published:** 2019-02-14

**Authors:** Sougat Misra, Carlos F. Moro, Marco Del Chiaro, Soledad Pouso, Anna Sebestyén, Matthias Löhr, Mikael Björnstedt, Caroline S. Verbeke

**Affiliations:** 1 Department of Laboratory Medicine, Division of Pathology F46, Karolinska Institutet, Karolinska University Hospital Huddinge, SE-141 86, Stockholm, Sweden; 2 Department of Clinical Intervention and Technology (CLINTEC), Center for Digestive Diseases, Karolinska University Hospital and Division of Surgery, Karolinska Institutet, Stockholm, 14186 Sweden; 3 Department of Clinical Pathology/Cytology, Karolinska University Hospital, Stockholm, SE-141 86 Sweden; 4 Tumour Biology Laboratory, 1st Department of Pathology and Experimental Cancer Research, Semmelweis University, Budapest, 1085 Ulloi ut 26. Hungary; 5 Institute of Clinical Medicine, University of Oslo, Postbox 1171 Blindern, Oslo, 0318 Norway; 6 Department of Pathology, Oslo University Hospital, Rikshospitalet, Postbox 4956 Nydalen, Oslo, 0424 Norway

## Abstract

Pancreatic ductal adenocarcinoma (PDAC) has a poor prognosis, which is mainly due to late diagnosis and profound resistance to treatment. The latter is to a large extent attributed to the tumor stroma that is exceedingly prominent in PDAC and engages in complex interactions with the cancer cells. Hence, relevant preclinical models of PDAC should also include the tumor stroma. We herein describe the establishment and functional validation of an *ex vivo* organotypic culture of human PDAC that is based on precision-cut tissue slices from surgical specimens and reproducibly recapitulates the complex cellular and acellular composition of PDAC, including its microenvironment. The cancer cells, tumor microenvironment and interspersed remnants of nonneoplastic pancreas contained in these 350 µm thick slices maintained their structural integrity, phenotypic characteristics and functional activity when in culture for at least 4 days. In particular, tumor cell proliferation persisted and the grade of differentiation and morphological phenotype remained unaltered. Cultured tissue slices were metabolically active and responsive to rapamycin, an mTOR inhibitor. This culture system is to date the closest surrogate to the parent carcinoma and harbors great potential as a drug sensitivity testing system for the personalized treatment of PDAC.

## Introduction

Pancreatic cancer is the fourth leading cause of cancer-related death in the West, and it is expected to rank second by 2030^1,2^. The reasons for the high mortality are late diagnosis and pronounced resistance to treatment^3^. There is thus a clear need for new, effective therapies.

The conventional steps in the process of drug testing rely significantly on the use of cell lines and xenograft-based or genetically engineered animal models^4^. Even if these models more or less faithfully recapitulate some of the features of human pancreatic cancer *in vivo*, there are serious limitations. These include the lack of the tumor microenvironment and the use of hosts from different species that may be immunocompromised. Accordingly, the average rate of successful translation from animal models to clinical cancer trials is less than 8%^5^, and the rate of implementation into clinical practice is even lower. In spite of the remarkable diversity of available test models, decades-old drugs are still the mainstay of clinical practice, especially for pancreatic cancer.

The tumor microenvironment is increasingly being identified as an important active component of pancreatic cancer that has pleiotropic interactions with and effects on the cancer cell population^6^. Of particular therapeutic importance is the fact that the stroma, which is characteristically prominent in pancreatic cancer, can compromise drug delivery, not only through mechanical hindrance but also by active scavenging of cytotoxic drugs^7^.

Several culture systems are available that allow preservation of tissue from human pancreas and pancreatic cancer for *ex vivo* testing, including a range of organotypic cultures of two or more cell types that form complex, organ-like structures^8–11^. Here we present an *ex vivo* culture system for precision-cut slices of human pancreatic cancer. This model offers a close approximation of the *in situ* tumor in the patient, as the slices contain all the constituent cell types and acellular components that are resident in pancreatic cancer in their original configuration. The aim of the study was to develop optimized culture conditions to keep precision-cut slices of human PDAC viable for at least 4 days, and to investigate whether structural and functional integrity of the constituting neoplastic and non-neoplastic tissues are compromised by identifying and characterizing temporal changes in key morphological features and protein expression patterns.

## Materials and Methods

### Patients and tissue samples

Fresh tumor tissue samples were collected from surgical resection specimens of chemonaïve primary resectable PDAC (n = 12; culture IDs OT1-OT12; OT referring to “organotypic”). Samples were collected at Karolinska University Hospital between 2014 and 2016. Clinicopathological characteristics are presented in Table 1.

**Table 1:** Clinicopathological data and culture conditions.

Culture ID	Age	Gender	Histopathological grade of differentiation	Stage*
OT1	68	Male	Moderate	pT3 N1
OT2	65	Female	Poor (non-gland forming)	pT3 N1
OT3	76	Male	Well	pT3 N0
OT4	75	Male	Poor	pT3 N1
OT5	74	Male	Well	pT3 N1
OT6	75	Male	Poor	pT3 N1
OT7	71	Male	Well	pT3 N1
OT8	76	Female	Poor	pT3 N1
OT9	62	Male	Well	pT3 N1
OT10	64	Male	Poor (non-gland forming)	pT3 N1
OT11	47	Female	Moderate to poor	pT3 N1
OT12	80	Male	Moderate	pT3 N1

*Stage – TNM classification for pancreatic cancer (7^th^ Edition).

The study was approved by the Regional Ethical Review Board, Stockholm (diary number 2012/1657-31/4). Written informed consent was obtained from all patients prior to surgery. All study methods were performed in accordance with the relevant guidelines and regulations.

### Preparation of precision-cut tissue slices

**Figure 1:**
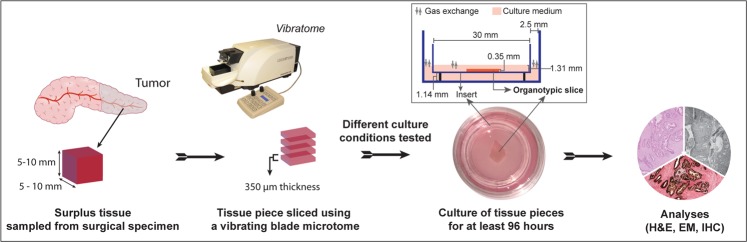
Diagram depicting the workflow of preparation, culturing and analysis of precision-cut tissue slices. Inset: A technical drawing (not to scale) showing the dimensions of different components of organotypic slice culture. In the adapted method, the membrane of the insert raised the deep aspect of the tissue slices approximately 1.14 mm from the bottom of the culture well. The tissue slices were submerged in medium (height ~1.31 mm), leaving ~1.0 mm of space for medium to cover the slices, thereby keeping the diffusion distance at a minimum. The space between the insert and the culture dish provided a surface area of ~255 mm^2^, allowing direct gas exchange between the ambient milieu and the culture medium.

Fresh resection specimens were received at the pathology laboratory within 20 minutes of surgical removal. A piece of tumor tissue (approximate dimensions 7 × 5 × 5 mm) was sampled by a specialist pancreatic pathologist (CSV, CFM) using a sterile scalpel. The tissue piece was sliced using a vibrating-blade microtome (VT1200S, Leica, Germany) equipped with a sapphire microtome blade. The tissue piece was fixed to the metal buffer tray with 3M Vetbond Tissue Adhesive and then submerged in ice-cold transport medium. Tissue was cut into 350 μm thick slices with the following operational settings: speed 0.05–0.15 mm/s; amplitude 1.30 mm. On average, the slicing procedure took 45 to 60 minutes and yielded between 12 and 17 slices per tissue sample. Using a thin brush, the tissue slices were transferred to a 60 mm sterile dish (BD Falcon, Thermo Fisher, Sweden) containing transport medium. The first slice was immediately fixed in formalin (control slice, time point 0 h) and embedded in paraffin for histomorphological evaluation. Subsequent slices were kept in ice-cold transport medium until completion of the slicing process. Each tissue slice was rested on an insert (0.4 µm pore size, 30 mm diameter, Millicell^®^, Millipore, Ireland) that was placed in a 35 mm culture dish (BD Falcon, Thermo Fisher, Sweden) containing 1.1 ml of complete culture medium. In addition, 0.55 ml of culture medium were added on top of the tissue slice to keep it submerged. A flow chart of the study procedure is shown in Fig. 1.

### Culture conditions

At the inception of the present study, we have carefully focused on establishing conditions that allow successful culturing of PDAC with good tissue viability in a reproducible manner and without the use of exogenous mitogenic factors and chemical inhibitors that may facilitate non-physiological cell survival and proliferation. Such an approach minimizes the influence of exogenous factors that can potentially alter the signaling circuitry of resident cells. At the outset of the study, basic culture conditions related to the choice of the culture medium, supplementation with antioxidants and use of an insert were systematically optimised by comparing parallel cultures, changing one variable at a time with 3 repeats (cfr. flowchart in Supplementary Fig. S1). In initial studies, DMEM medium supplemented with 10% FCS was compared with CMRL 1066 without glutamine supplemented with 2.5% human serum (type AB, Sigma-Aldrich, Germany) for culturing the slices. As preliminary experiments indicated better viability of tissue slices in CMRL medium, the latter was used in all subsequent experiments. CMRL medium was supplemented with cell culture grade 25 mmol/L HEPES (Gibco, Thermo Fisher, UK), 1 mmol/L sodium pyruvate (Gibco, Thermo Fisher, UK), 3 nmol/L zinc sulfate (ZnSO_4,_ Sigma-Aldrich, Germany), 1X insulin-transferrin-sodium selenite solution (Gibco, Thermo Fisher, UK), 2.5% human serum AB, 1X PenStrep (Gibco, Thermo Fisher, UK), and 100 nmol/L diphenyl diselenide (Sigma-Aldrich, Germany). Use of antioxidants (N-acetylcysteine or pycnogenol) in CMRL media yielded no additional survival advantages. However, we used diphenyl diselenide as a culture supplement based on our preliminary findings that this compound exhibits antioxidant-like properties when used at a low concentration (own unpublished data). Inserts were used, as initial optimization studies indicated better viability of tissue slices that were supported, and in particular, easier handling of the tissue slices during exchange of medium with reduction of the risk of inflicting mechanical damage to the tissue.

Following previous studies on precision-cut slice cultures of human pancreas and pancreatic cancer that used a highly oxygenated environment (90–95%) to prevent oxygen deprivation in tissue slices devoid of blood circulation^12,13^, tissue slices were initially cultured at 37 °C in a humidified incubator with 41% oxygen tension (OT1-OT9; Supplementary Fig. S1). Every 24 h, culture medium was exchanged with fresh medium that had been pre-conditioned for at least 1 h in the incubator. Slices were maintained in culture for at least 96 h. Following success in maintaining tissue slices at 41% oxygen tension and detailed characterization of various aspects of the cancer and microenvironment (cfr. below), a subsequent series of experiments (n = 3; OT10-OT12) was undertaken in which paired tissue slices obtained from the same surgical specimen were cultured under both normoxic (21% O_2_) and hyperoxic (41% O_2_) conditions to assess whether such difference in O_2_ tension promotes or compromises tissue viability, integrity, and cell proliferation. Ice-cold Hank’s Balanced Salt Solution (HBSS, with Ca^2+^ and Mg^2+^) supplemented with 1X Pen-Strep solution and 500 nmol/L diphenyl diselenide was used as transport medium in all experiments.

### Qualitative and quantitative histomorphological assessment

Duplicate tissue slices were harvested from culture every 24 h, formalin-fixed, paraffin-embedded, and sectioned (4 μm thick) for histological assessment. Sections were cut horizontally, except for those related to the testing of the effect of oxygen tension, which were cut vertically. Tissue processing, sectioning, and staining (hematoxylin-eosin and immunohistochemistry) was performed by the accredited routine histopathology laboratory at Karolinska University Hospital. Hematoxylin-eosin (H&E) stained tissue sections were evaluated to confirm the presence of PDAC and to assess the grade of tumor differentiation^14^. Tumors were subsequently analyzed according to the following groups: well, moderate to poorly, and non-gland forming poorly differentiated. The latter were analyzed as a separate group because cancer cells exhibited marked differences in their growth pattern and proliferation compared to gland forming tumors, as detailed in Results. Digital morphometric histological analyses were performed on whole slide images (Hamamatsu NanoZoomer slide scanner and Hamamatsu NDP.view, version 2.6, for manual annotation of tissue viability and cell outgrowth; 3DHistech Pannoramic SCAN II slide scanner, 3DHistech Pannoramic Viewer version 1.15, 3DHistech CaseViewer version 2.1).

### Tissue viability and outgrowth measurement

The usual viability assays quantify cellular ATP levels, mitochondrial activity or activation of proteins implicated in specific modes of cell death. The heterogeneity of pancreatic cancer tissue due to the varying admixture with non-neoplastic stroma and pancreatic parenchyma results in variability in the read outs of such assays that is difficult to interpret. We therefore chose to identify and quantify morphological changes that point at cellular damages in the various cell populations. Areas of necrosis or apoptosis were identified and manually annotated in the digital slides in a blinded manner. Their overall extent was recorded as the percentage of the entire tissue area. In addition, the zonal distribution in the peripheral, intermediate and central regions of the tissue slices were calculated using R and rgeos^15^ package for spatial data analysis. Each zone had a width of one-third of the tissue slice diameter. Outgrowths of cancerous and non-neoplastic epithelial cells onto the tissue slice surface were manually annotated, and the length of surface they covered was recorded as the percentage of the total perimeter for each tissue slice. Global cell outgrowth was calculated as the sum of both cancerous and non-neoplastic outgrowths. Findings were averaged over duplicate slices from each culture sampled at various time points (0 h, 24 h, 48 h, 72 h, and 96 h).

### Transmission electron microscopy (TEM)

As an additional method to study cell and tissue integrity, TEM was used to detect morphological changes at the ultrastructural level at 0 h and on duplicate samples (OT-culture IDs 5, 11, and 13) at 24 h and 72 h. Briefly, slices were washed twice with PBS and fixed overnight in 2.5% glutaraldehyde in 0.1 M phosphate buffer, pH 7.4 at 4 °C. Slices were post-fixed for 2 h in 2.0% osmium tetroxide in 0.1 M phosphate buffer, pH 7.4 at 4 °C, subsequently dehydrated in ethanol followed by acetone, and embedded in LX-112 (Ladd, Burlington, Vermont, USA). Ultrathin sections (50–60 nm) from regions containing tumor were cut using a Leica Ultracut UCT/Leica EM UC 6 (Leica, Wien, Austria). Sections were contrasted with uranyl acetate followed by lead citrate and examined in a Hitachi HT7700 electron microscope (Tokyo, Japan) at 80 kV. Digital images were acquired using a Veleta camera (Olympus Soft Imaging Solutions, GmbH, Münster, Germany).

### Immunohistochemical analysis

Immunohistochemical staining was performed using a Leica BOND III automated immunostainer with a panel of antibody markers for the identification of epithelial cells, stromal cells and immune cells that together form the tumor microenvironment in pancreatic cancer: CK18 (n = 2) for epithelial cells, CK19 (n = 5) and CA19-9 (n = 2) for ductal-type epithelial cells, and trypsin (n = 3) for pancreatic acinar cells; p53 (n = 5), SMAD4 (n = 6), and maspin (n = 2)^16^ to discriminate between cancerous and non-neoplastic cells; and Ki67 (n = 7) for assessment of proliferative activity. In the absence of a single representative marker for pancreatic cancer stroma, a panel of markers selected from the literature was used to analyze the stroma in the tissue slices (unless otherwise stated, n = 2: OT5, OT7): vimentin (n = 4) for mesenchymal cells and in particular stromal fibroblasts; α-smooth muscle actin (α-SMA)^17^, a marker of activated pancreatic stellate cells and cancer-associated fibroblasts; D2-40 (podoplanin)^18^, a further stromal marker expressed in pancreatic cancer-associated fibroblasts; CD34^19^, a marker of fibrocytes in non-neoplastic pancreas, whose expression is decreased in pancreatic cancer; and caldesmon for smooth muscle cells. Immune cells were analyzed with CD3, CD20, and CD68 (n = 2) for T-cells, B-cells, and macrophages, respectively. Antibodies were combined by multiplex immunohistochemistry (CK18/α-SMA, Ki67/vimentin, p53-CD34/caldesmon-CK19, p53-D2-40/caldesmon-maspin, CD3/CD20, and CD68/CA19-9) for better visualization of the spatial relationship between the various cell populations. For details of the antibodies used in this study, see Supplementary Table S1. The stromal and immune cell markers (n = 2) and the tumor epithelial cell marker CK19 (n = 2, OT2, OT10) were quantified using QuPath v1.3^20^ and recorded as the percentage of stained cell area with respect to the entire tissue area.

### Measurement of proliferative activity

Proliferation was assessed by immunostaining for Ki67 on all slices (control and at the four time points) from seven cultures. The latter comprised at least two carcinomas for each grade of histological differentiation: poorly non-gland forming (OT2, OT10), moderately to poorly (OT4, OT11, OT12), and well differentiated (OT5, OT7). The Ki67-index, i.e. the percentage of Ki67-positive cancer cells, was assessed in hot-spot fields (median = 3, range = 1–4), which were photographed at 200X magnification using digital slide viewer and manually quantified using ImageJ version 1.50i, National Institutes of Health U.S. The Ki67-index was assessed separately in the following tissue compartments: (i) cancer cells within the tissue slices and (ii) cancer cells and (iii) non-neoplastic epithelial cells growing onto the surface of the tissue slices. Findings for the respective tissue compartments were averaged over the duplicate slices in each culture and at each time point.

### Measurement of pS6 (mTOR pathway)

A total of five PDAC cultures, comprising one tumor of each differentiation grade (OT2, OT7, OT12) and three matched cultures under normoxic and hyperoxic conditions (OT10, OT11, OT12), were immunohistochemically investigated for phosphorylation of ribosomal protein S6 (pS6), downstream of mTOR metabolic pathway, at all 5 culture time points (0 h–96). In each tissue slice, the optical density of pS6 staining in the cancer cells was quantified in a hot-spot area containing at least 200 cancer cells using QuPath v1.3. Cancer cells were discriminated from stroma, immune and non-neoplastic epithelial cells by creating a tailored machine learning Random Trees classifier after watershed cell detection.

### Measurement of tissue oxygenation and tissue viability under normoxic and hyperoxic conditions

Oxygenation in tissue slices cultured at different oxygen tensions (hypoxic, less than 21%; normoxic, 21%; and hyperoxic, 41%) was assessed using the pimonidazole dye (final concentration 250 µM, HypoxyprobeTM-1 kit, Hypoxyprobe, Inc, USA) in paired tissue slices obtained from 3 different donor samples (OT10-OT12). To create hypoxic conditions, complete medium without serum was bubbled with nitrogen for 120 minutes. Subsequently, human serum AB (2.5%) and pimonidazole were added to the medium. To minimize gas exchange, tissue slices were covered with excess medium, leaving minimal air space between the medium and lid of the dish. The latter was subsequently wrapped with parafilm and placed in an incubator with normal O_2_ tension. To create hyperoxic or normoxic ambient conditions, complete culture medium was conditioned for 1 h in an incubator with the O_2_ level set at 41% or 21%, respectively. Following 2 h of incubation in the respective conditions, slices were collected, fixed and embedded in paraffin as described above. Immunohistochemial staining for pimonidazole was performed on vertically sectioned tissue slices to study the extent of hypoxia across the entire thickness of the precision-cut slices, i.e. from the tissue surface facing the air-medium interphase to the surface facing the medium/insert. For comparison, CAIX (OT12) was used as an additional marker for hypoxia^21^.

Tissue viability and cancer outgrowth on the slice surface under normoxic and hyperoxic conditions were compared by morphometric analysis on H&E-stained sections.

### Statistical analyses

We employed non-parametric statistical tests to analyze all the data, because the sample size was small and insufficient to determine the exact distribution of the data. Comparisons of quantitative data of tissue viability, cell outgrowth, and proliferative activity were performed using either the Friedman test or Wilcoxon matched paired signed-rank test, as applicable. Dunn’s test was employed for multiple comparison purposes, unless stated otherwise. A p-value of <0.05 was considered statistically significant. All the data analyses were performed using GraphPad Prism, Version 6 (Prism Inc., USA) and R (version 3.4.3).

## Results

### Tissue handling provides representative samples of pancreatic cancer

All samples taken according to the study protocol contained pancreatic cancer tissue in amounts that were sufficient to perform the planned investigations. The tissue slicing procedure usually yielded slices of sufficient quality to allow further culturing and analysis, except for tissue pieces with high fat and mucin content which could not withstand the pressure created by the moving blade. Incomplete or fragmented tissue slices were not used for culturing. The median number of tissue slices obtained per tissue sample taken from the surgical specimen was 14 (range: 12–17).

Following initial trimming, a single 350 μm thick tissue slice yielded over 60 tissue sections by horizontal serial sectioning. The median area of horizontal tissue sections was 31.9 mm^2^ (range: 20.1–57.9 mm^2^).

### Tissue damage occurs early but remains stable thereafter and affects mainly the outer part of the tissue slice

**Figure 2:**
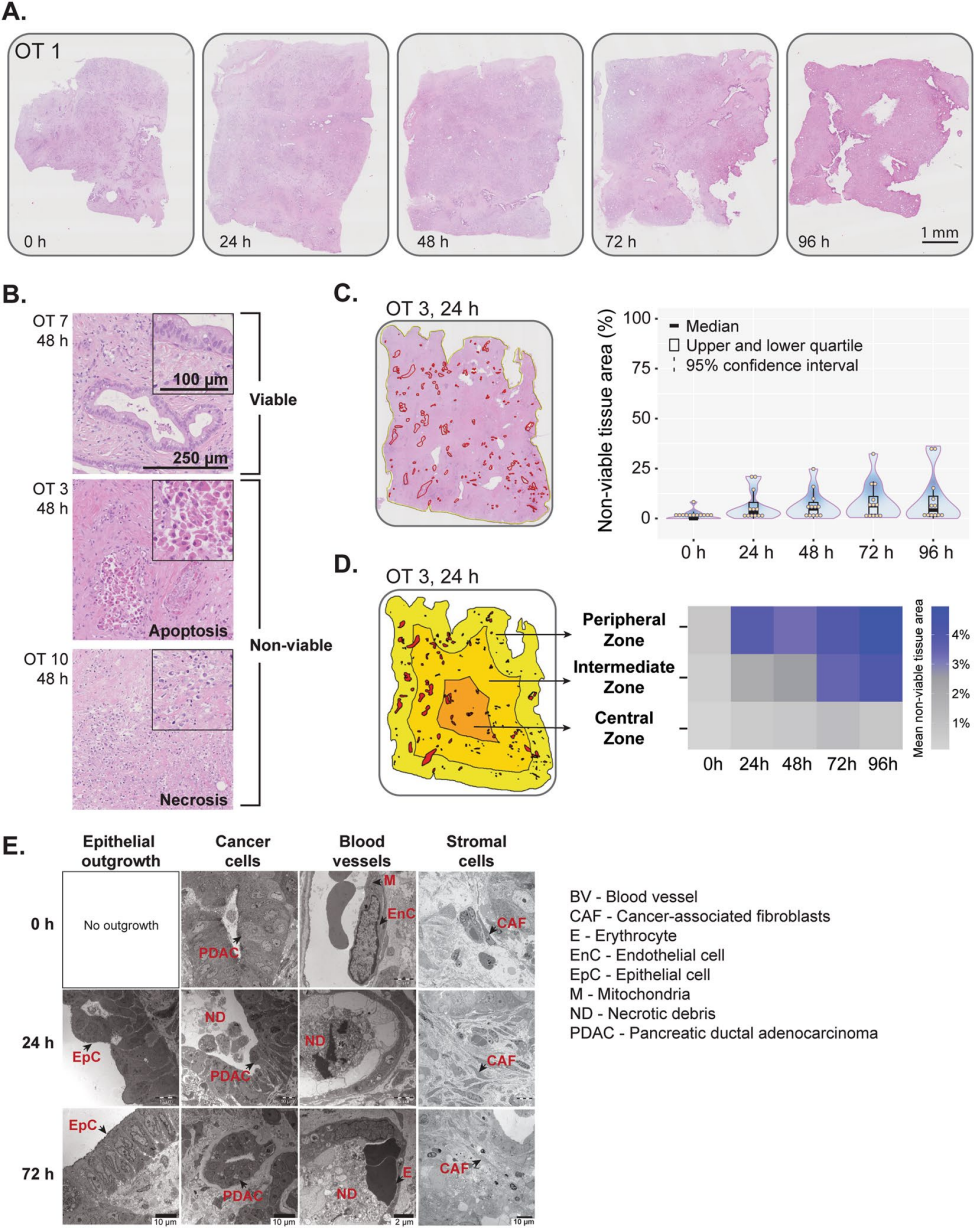
Tissue viability and structural integrity. (**A**) Representative overview images of H&E-stained whole tissue slices. See Figure S1 for high magnification images. (**B**) Representative H&E-stained tissue slices showing viable tissue and foci of apoptosis and necrosis. (**C**) Left panel, Digital annotation of non-viable tissue areas within the slice. Red annotated areas indicate non-viable tissue. Right panel, Quantification of non-viable tissue areas during culture time (n = 12; Friedman test followed by Dunn’s multiple comparison; p > 0.05). In the violin plot, the central band, box and whiskers represent median value, interquartile range, and maximum and minimum values, respectively. (**D**) Schematic diagram showing the annotation of a whole slice into 3 separate zones - peripheral, intermediate and central, each with a width of one-third of the tissue slice diameter. The heatmap shows spatial distribution of mean non-viable tissue areas (%) in the 3 zones with respect to culture time. The scale indicates mean values. (**E**) Representative transmission electron photomicrographs (n = 3) of control (0 h) and cultured slices (24 h and 72 h) depicting morphological alterations of epithelial outgrowth, cancer cells, blood vessels and stromal components during culture.

Light microscopic evaluation revealed overall good morphological preservation of the tissue slices during the entire culture period (up to 96 h, Fig. 2A, Supplementary Fig. S2). Necrotic and apoptotic cell death were observed in discrete areas of the tissue slices (Fig. 2B**)** within the first 24 h of culture (statistically non-significant compared to control tissue at 0 h), while no further tissue damage was observed during subsequent 72 h of culture (Fig. 2C, right panel). Next, we interrogated if there was any spatial difference in the occurrence of tissue damage. Separate quantification of the extent of necrosis and apoptosis in the central, intermediate and peripheral one-third of the tissue slice area showed that the latter incurred the highest tissue damage followed by the intermediate zone (Fig. 2D**)**. When comparing the spatial distribution of non-viable tissue areas at individual time points, both the peripheral and intermediate zone showed significantly higher tissue damage compared to the central zone at all time points except for 0 h (Supplementary Fig. S3A**)**. Temporal distribution data showed significant loss of viable tissue both in the peripheral and the intermediate zone within the first 24 h, without further exacerbation of tissue damage over time (Supplementary Fig. S3B**)**. A statistically significant, but marginal loss of viability was recorded in the central zone at a late stage during culturing (72 h, Supplementary Fig. S3B). Findings were similar throughout the full thickness of precision cut slices, i.e. there was no significant difference in either extent of tissue damage or zonal distribution between tissue sections that stemmed from closer to the surfaces facing the air or insert and those that represented central parts of the cultured tissue slices. Examination of the ultrastructural morphology by TEM showed variable structural integrity of different cellular components **(**Fig. 2E**)**. At 0 h, two of the cases showed in discrete areas pallor of the mitochondrial matrix in cancer cells, indicating loss of mitochondrial membrane integrity (data not shown). Occasional small collections of cellular debris were seen within cancer glands. Endothelial cells were often swollen, but endothelial cell-to-cell contact was intact. Stromal cells including fibroblasts located within the tumor-associated stroma, were generally well preserved, and only in one case, discrete necrotic foci were present. After 72 h culture, most cell populations, including the cancer cells, maintained ultrastructural integrity, with the exception of occasional cancer glands and small vessels that contained necrotic debris in the lumen, showed extravasation of erythrocytes and disrupted cell-to-cell contact of the endothelial lining. Overall, at an ultrastructural level, damage was mainly limited to the endothelial and cancer cells and ranged from minor subcellular changes to the presence of focal cellular necrosis.

### Expression of markers that characterize various cell populations in cancer- and pancreas-associated stroma is retained in culture

**Figure 3:**
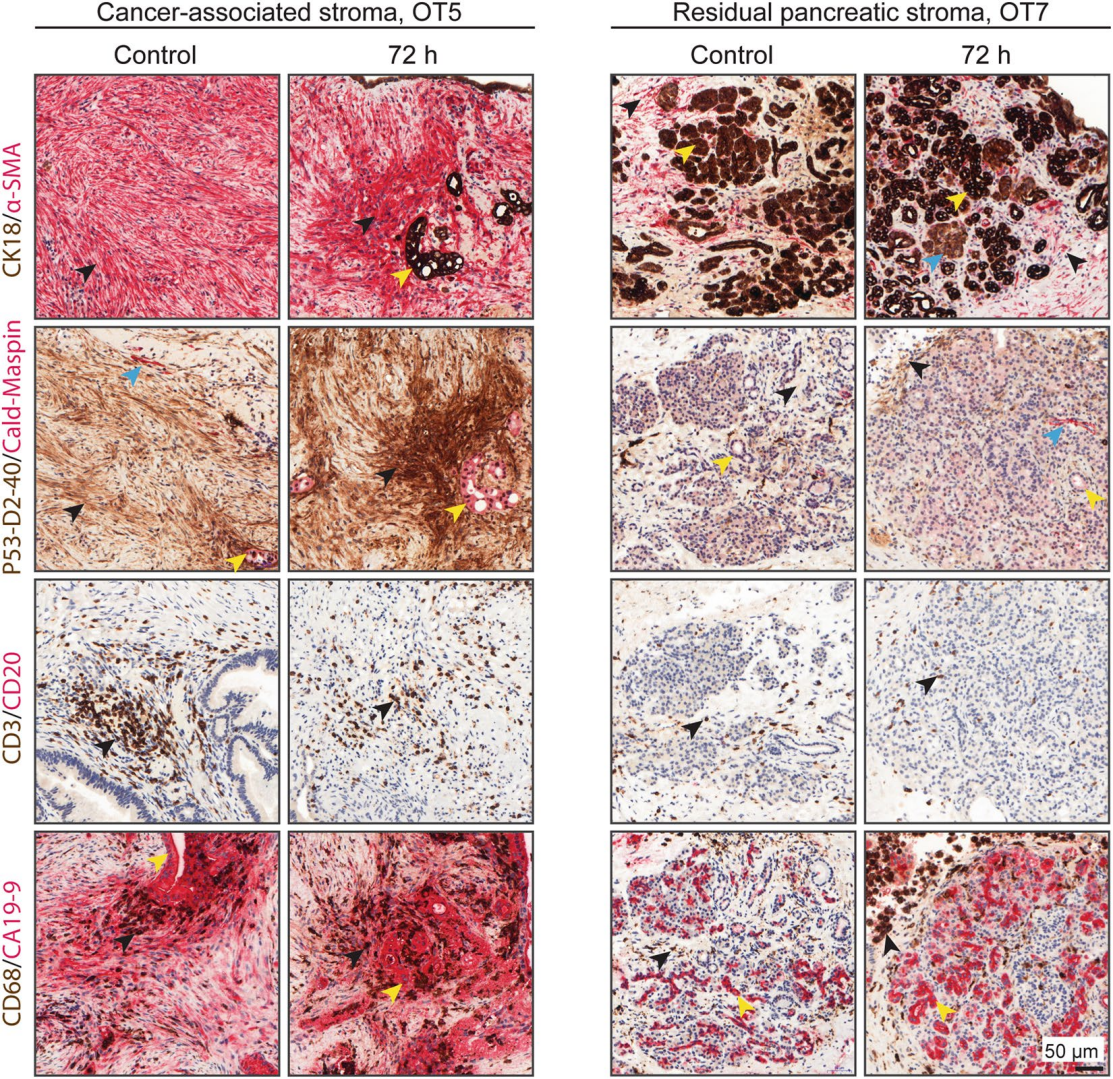
Immunohistochemical profiles of stromal cells. Both cancer-associated and residual pancreatic stroma retained the expression of their characteristic immunohistochemical markers over the course of the culture period. Cancer-associated stroma: Cancer-associated fibroblasts are stained with α-smooth muscle actin (α-SMA) and D2-40 (black arrows). Sparse smooth muscle cells are positive for caldesmon (blue arrows). Clusters of PDAC cells are positive for CK18, maspin, P53, and CA19-9 (yellow arrows). Note that there is also CA19-9 immunoreactivity in the stroma surrounding PDAC cells. CD3-positive T-cells and CD68-positive macrophages are also present in the stroma (black arrows). Residual pancreatic stroma: Myofibroblasts in residual, partially atrophic pancreatic lobuli are positive for α-SMA (black arrows). Acinar, ductal (yellow arrows), and endocrine (blue arrow) epithelial cells are stained with CK18. Lobular stroma is negative or minimally positive for D2-40 (black arrows). In contrast to PDAC cells, non-neoplastic ducts are negative for both maspin and P53 (yellow arrows). Sparse CD3-positive T-cells and variable numbers of CD68-positive macrophages are also present (black arrows). Small, non-neoplastic ductal structures are positive for CA19-9 (yellow arrows).

As the stroma is a key component of the microenvironment, both the cancer-associated stroma and the stroma of residual pancreatic parenchyma were characterized at 0 h and 72 h based on immunohistochemical detection of cellular markers for the various constituting cell populations. At both time points, cancer-associated stroma was positive for vimentin, α-smooth muscle actin (α-SMA) and D2-40, while it was negative for CD34. In contrast, residual pancreatic stroma was positive for vimentin, CD34, and partially for smooth muscle actin, while D2-40 staining was negative or minimal (Fig. 3, Supplementary Fig. S4A). Quantification for the various markers revealed marginal differences between both time points (Supplementary Fig. S4B). Similarly, CD3+ T-lymphocytes and CD68+ macrophages were preserved during culture, both in cancer-associated and residual pancreatic stroma. Taken together, analysis of the expression of cellular markers shows that the stroma associated with either the cancer or residual pancreatic parenchyma retains its characteristically heterogeneous cellular composition.

### Tumor morphology remains unaltered during culture

**Figure 4:**
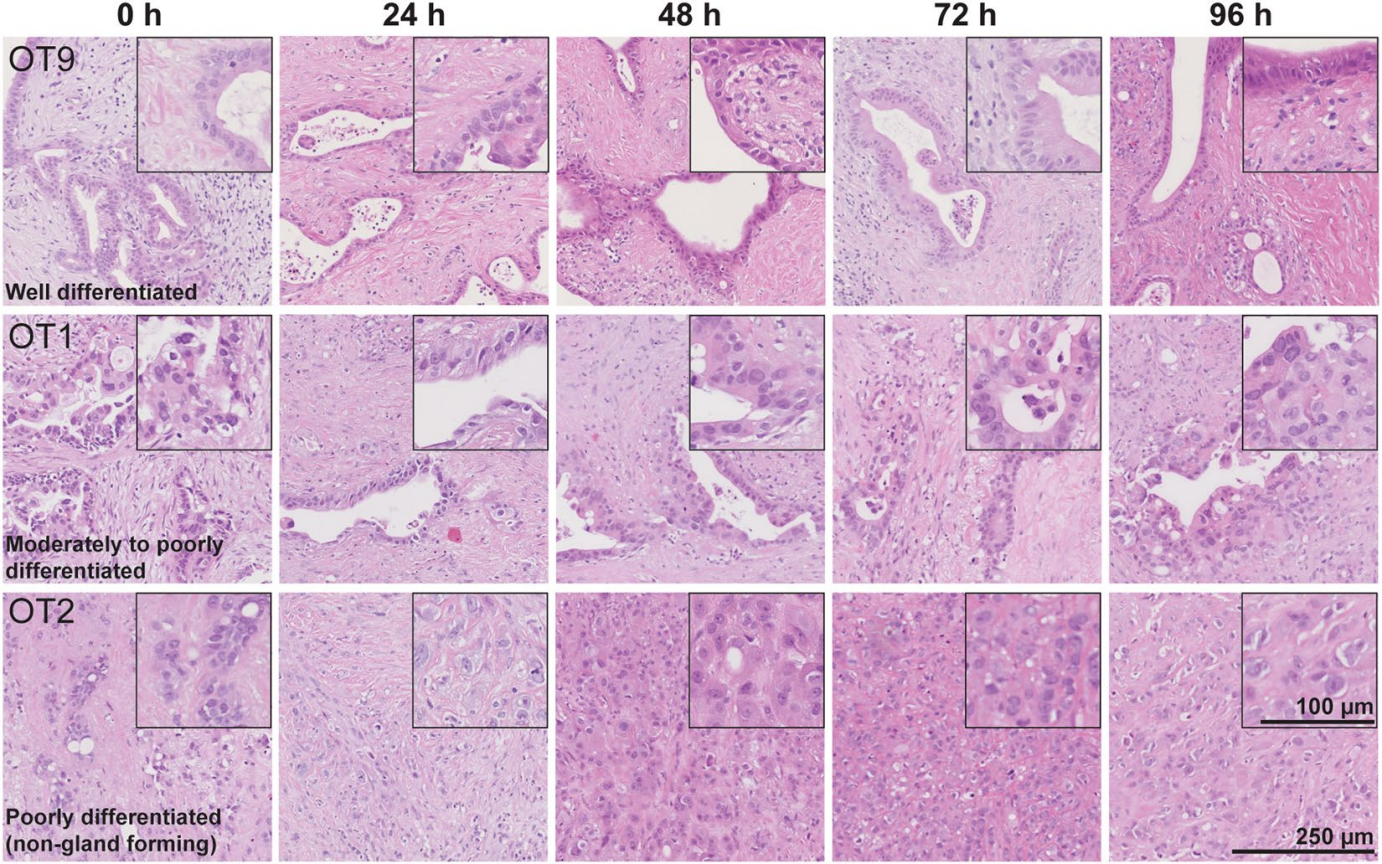
Tumor morphology and grade of differentiation. PDACs retained their histological and cytological features and grade of differentiation during the entire culture period (H&E staining).

Each of the cultured adenocarcinomas retained its characteristic histological and cytological features and grade of differentiation throughout the culture period (96 h) without any change as compared to the control sections taken at time point 0 h **(**Fig. 4**)** and the source tissue.

### Cancer cells more than non-neoplastic duct epithelium grow onto the tissue surface

**Figure 5:**
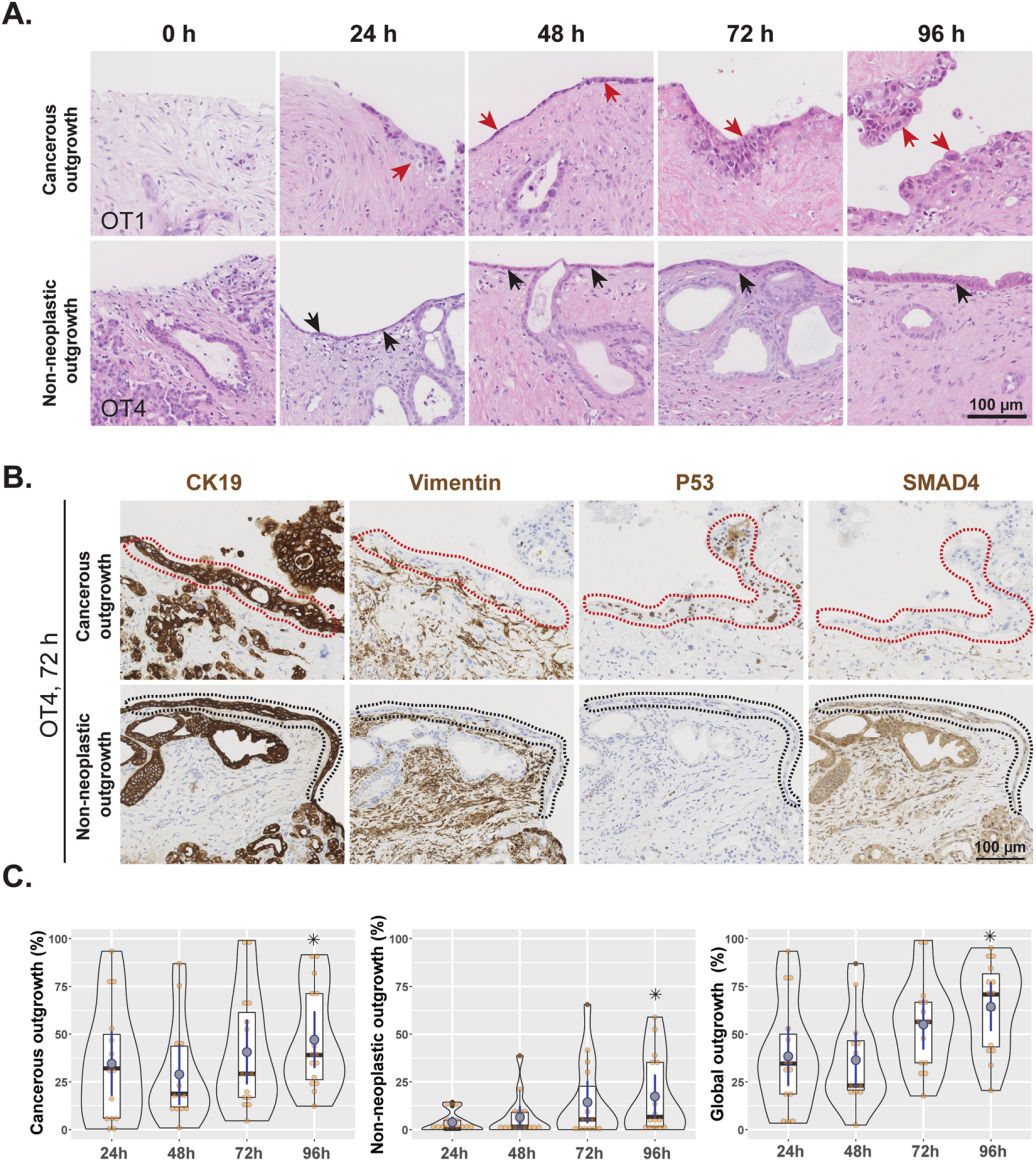
Cellular outgrowth onto the tissue surface. (**A**) Representative H&E-stained images showing cancerous (red arrows) and non-neoplastic (black arrows) cell outgrowth at different time points (0 h to 96 h). (**B**) A representative example of tissue slices with areas of cancerous and non-neoplastic cell outgrowth. Immunohistochemical staining showed that both types of outgrowth were positive for CK19 and negative for vimentin, indicating that outgrowth is of ductal-epithelial origin. Cancerous cell outgrowth (red dotted areas) was positive for P53 and negative for SMAD4, in contrast to the non-neoplastic one (black dotted areas), which was negative for P53 and positive for SMAD4. (**C**) Comparative analysis of cancerous, non-neoplastic, and global outgrowths at the indicated time points (duplicate slices from each culture, n = 8; Friedman test followed by Dunn’s multiple comparison, p ≤ 0.05; *indicates significant difference compared to 24 h time point). Time point 0 h was omitted from analysis due to the absence of any outgrowth.

Histological examination of cultured tissue slices revealed the gradual appearance of a continuous layer of cohesive cells on the surface of the tissue slices. Cellular outgrowth was absent in control slices (0 h) in all cases, but became evident at 24 h and increased steadily to cover most of the slice surface by 96 hours of culture (Fig. 5A). Light microscopic and ultrastructural morphology of the outgrowing cells as well as their immunohistochemical profile (CK19 positive and vimentin negative) was consistent with that of ductal epithelial cells (Fig. 5B). However, their cytomorphological features were not uniform. In some areas, the cells grew in a single row, were devoid of cytological atypia, resembled normal duct epithelium and were often found in direct continuity with transected pancreatic ducts at the edge of the tissue slices. Outgrowth was dominated by flattened cells at the beginning of culture, while cells became progressively taller and cylindrical in shape towards the end of the culture period. These findings were indicative of gradual outgrowth and maturation of the non-neoplastic ductal epithelium (Fig. 5A).

In other areas, cells showed a varying degree of cytological atypia and grew in irregular tufts, indicating that these were outgrowths of the cancer cell population onto the tissue surface. This could be confirmed immunohistochemically based on overexpression of P53 (OT2, 4, 6, and 7) and/or negativity for SMAD4 (OT4, 5, and 7) in tumor cells growing on the tissue surface (Fig. 5B) in cancers that showed aberrant P53 and/or SMAD4 expression in the entirety of the cancer cell population within the tissue slice.

Quantification data from 12 cultures showed that cancerous cell outgrowth was more extensive than that of non-neoplastic epithelial cells (Fig. 5C). Both cancerous and non-neoplastic epithelial outgrowths were significantly more extensive at 96 h than at 24 h. A similar trend was recorded for the global, i.e. combined cancer and non-neoplastic outgrowth at 96 h versus 24 h. These findings suggested the involvement of active tissue repair and proliferation in the cultured slices and therewith imply good structural and functional preservation over the course of culture period.

**Figure 6:**
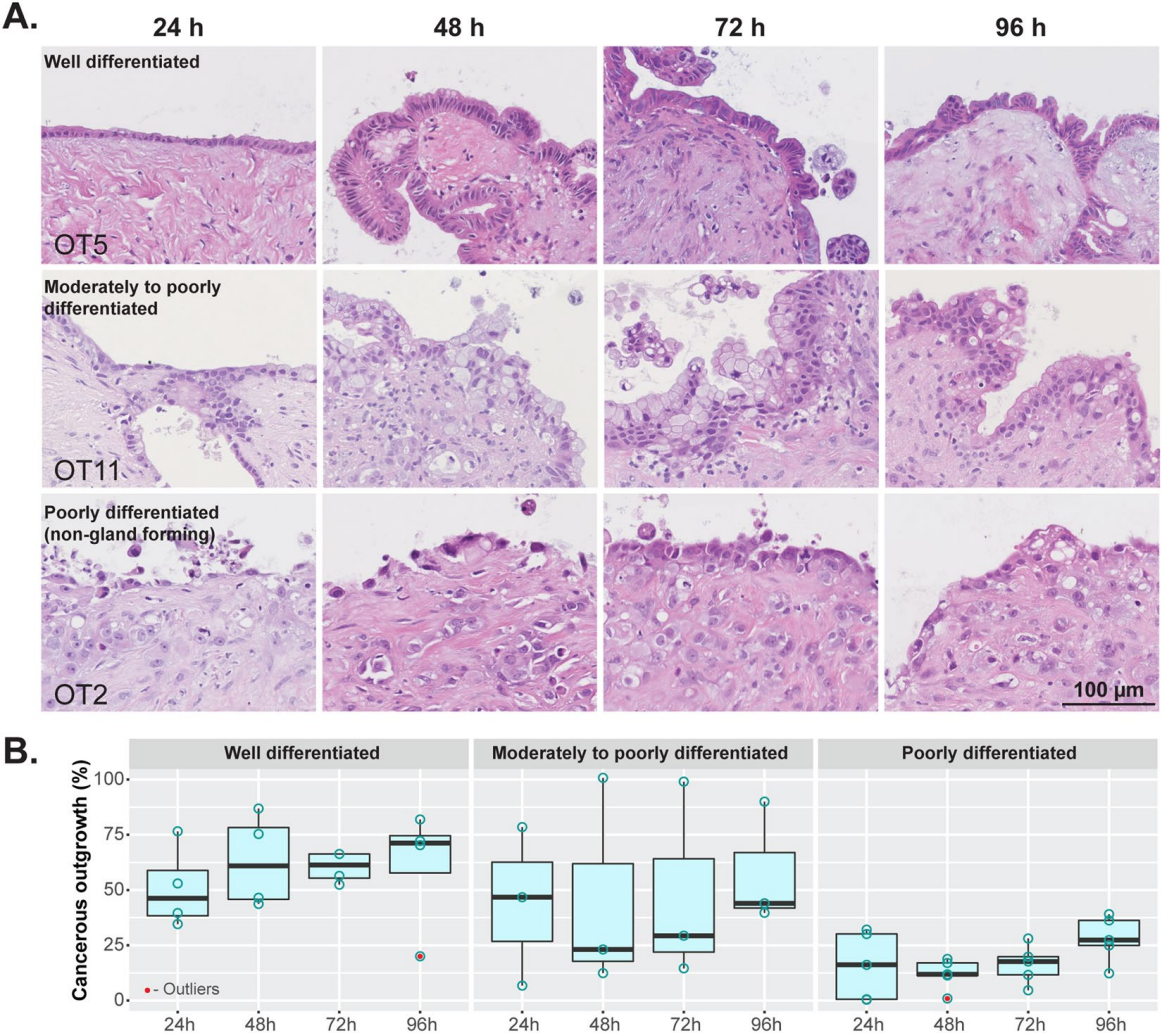
Cellular outgrowth and grade of tumor differentiation. (**A**) Well and moderately to poorly differentiated PDACs formed a continuous cell layer with foci of stratification, limited cytological atypia and varying degrees of loss of polarity. Non-gland forming poorly differentiated PDACs showed a patchy and discohesive cell outgrowth with marked cytological atypia (H&E staining). (**B**) Differentiation grade-wise quantification of outgrowth of cancerous cells at different time points (n = 3–5; Friedman test followed by Dunn’s multiple comparison, p > 0.05).

Cancer cell outgrowth maintained the characteristic cytomorphological features related to the grade of differentiation of the tumor within the tissue slices (Fig. 6A). Well differentiated tumors formed a continuous cell layer with occasional foci of stratification. Cell outgrowth from moderately differentiated tumors showed prominent cell stratification and loss of polarity. Non-gland forming poorly differentiated tumors, which showed a discohesive growth pattern in the stroma of the tissue slice, generated a discontinuous cell outgrowth that tended to converge as a patchy cell layering by the end of culture time. Furthermore, the extent of the cellular outgrowth relative to the perimeter of the tissue slice increased with better differentiation grade. By the end of culture time (96 h), the median percentage of the slice edge that was covered by cancer cell outgrowth was: poorly differentiated, non-gland forming carcinomas, 27.3% (range 12.3–39.0%); moderately differentiated, 43.9% (range 39.6–90%); and well differentiated, 71.2% (range 20.0–81.9%) (Fig. 6B).

In well-to-moderately differentiated cancers, the number of tumor cells within the tissue slice gradually declined up to 96 h culture, while conversely, the number of cancer cells on the surface of the slice increased to become the more prominent component at 96 h. In contrast, one of the non-gland forming, poorly differentiated carcinomas (OT2) grew steadily within the stroma, such that by the end of culture time a major part of the tissue slices was occupied by the proliferating cancer cells (Supplementary Fig. S5).

### Proliferative activity varies between tumors with different grades of differentiation

**Figure 7:**
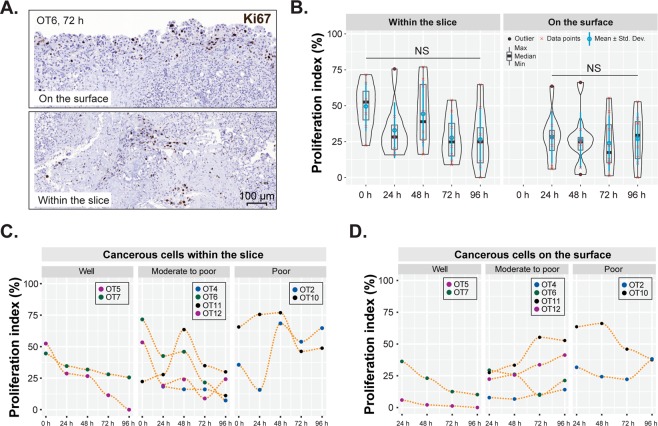
Quantification of cancer cell proliferation. (**A**) Representative examples of Ki67 positivity on the surface (cell outgrowth) and within the tissue slices. (**B**) Comparison of the proliferation index of cancerous cells within and on the surface of slices at different time points. (n = 8; Friedman test followed by Dunn’s multiple comparison, p > 0.05. (**C**,**D**) Proliferation index of PDACs with different grades of differentiation, growing inside the slice and on the slice surface. Poorly differentiated cases refer to non-gland forming tumors.

The Ki67-index was assessed at all time points in at least two samples of each grade of histological tumor differentiation. The cancer cells continued proliferating during culture, both inside the slice and on its surface (outgrowth) (Fig. 7A,B). Overall, proliferation was higher in poorly differentiated PDACs and in cancer cells growing inside the slices than those on the slice surface (Fig. 7B–D). During culture time, proliferative activity remained fairly steady in poorly differentiated tumors, but tended to decrease in well and moderately differentiated cases, especially in the cells growing inside the tissue slices.

Proliferative activity in the outgrowth of non-neoplastic epithelial cells (median Ki67-index: < 1% at 24 h, 6% at 72 h, 5% at 96 h) was much lower than in the cancer cells (median Ki67-index: 31% at 24 h, 17% at 72 h, 26% at 96 h; p ≤ 0.0001) and remained stable over time.

### *Ex vivo* culture induces increased metabolic activity which then remains stable during culturing time

**Figure 8:**
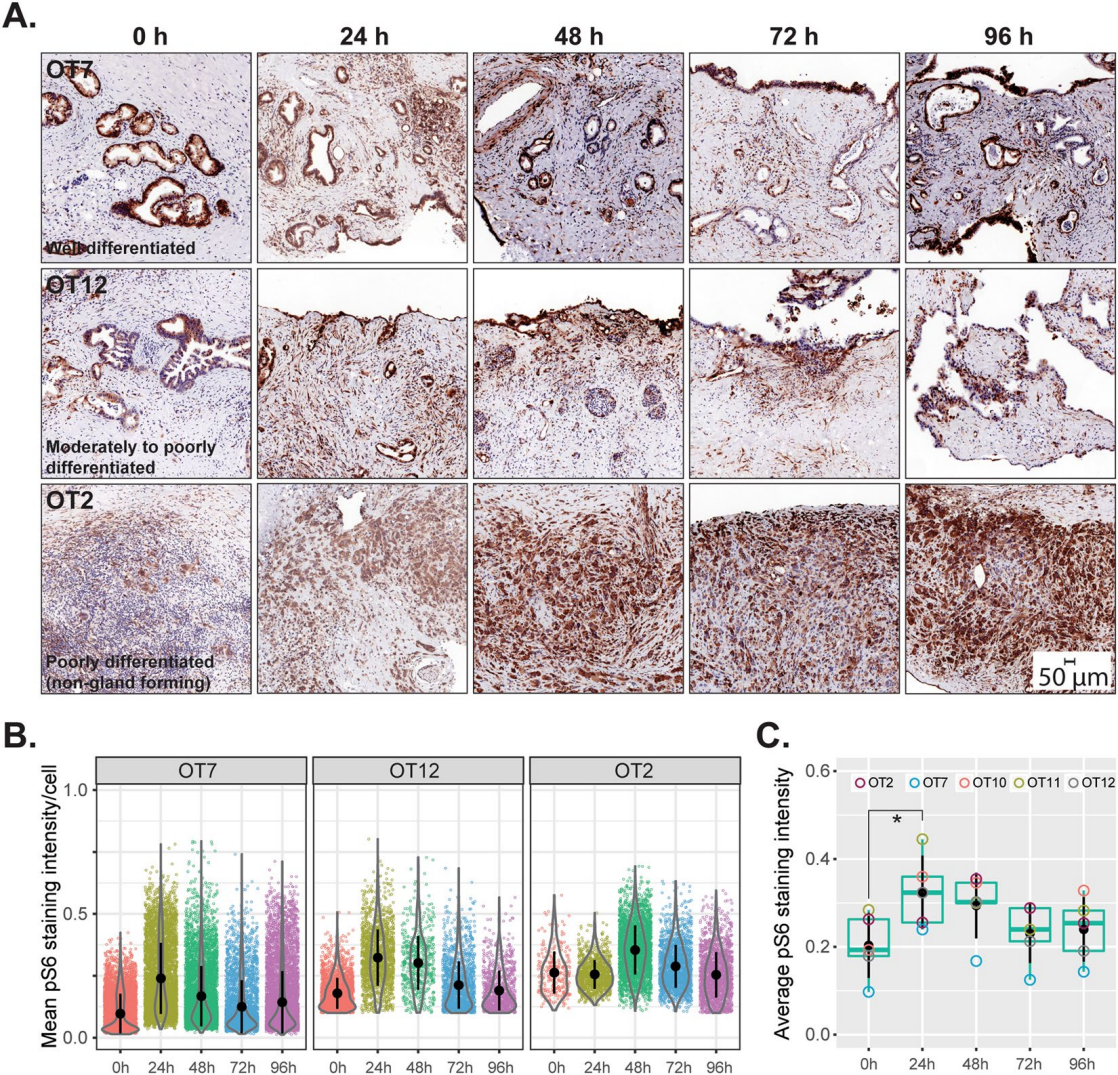
Metabolic activity. (**A**) Phosphorylated (Ser235/236) S6 ribosomal protein was used as a marker for the activity of mTOR, a master regulator of cellular metabolism. Positivity for S6 phosphorylation in all tissue slices at all time points indicates continued metabolic activity. (**B**) Quantitation of pS6 staining intensity in cancerous cells at different time points from the cases depicted in (**A**). (**C**) Comparative assessment of metabolic activity (average pS6 staining intensity, n = 5) of cancerous cells in cultured slices over the entire duration of culture (Friedman test followed by Dunn’s multiple comparison; p ≤ 0.05). * indicates significant difference between the given time points.

The mTOR pathway is a central regulator of cellular metabolism and growth. To investigate metabolic function in the tissue slices, we used phosphorylation of ribosomal protein S6 (pS6), downstream of mTOR, as a marker of metabolic activity. Highly metabolically active duodenal crypt cells were used as positive control for pS6 staining (Supplementary Fig. S6A). The cancer cells and cancer-associated stromal cells comprised the main cell populations that were positive for pS6 (Fig. 8A). The average levels of pS6 were higher at 24 h when compared to the non-cultured slices (0 h), and remained stable at later time points (Fig. 8B,C). To validate our findings, the cultured slices were treated with rapamycin (50 nM) for 72 h following an initial recovery period of 24 h. Rapamycin treatment resulted in a substantial reduction of pS6 levels compared to untreated control slices (Supplementary Fig. S6B). This series of experiments demonstrated that cultured slices were metabolically active - albeit at a higher level than in non-cultured control tissue - and that they successfully recapitulated the pharmacological inhibition of a signaling molecule in the pathway that is a central regulator of metabolic activity.

### Normoxic and hyperoxic culture conditions do not significantly affect tissue viability, markers of hypoxia, metabolic activity and proliferation

**Figure 9:**
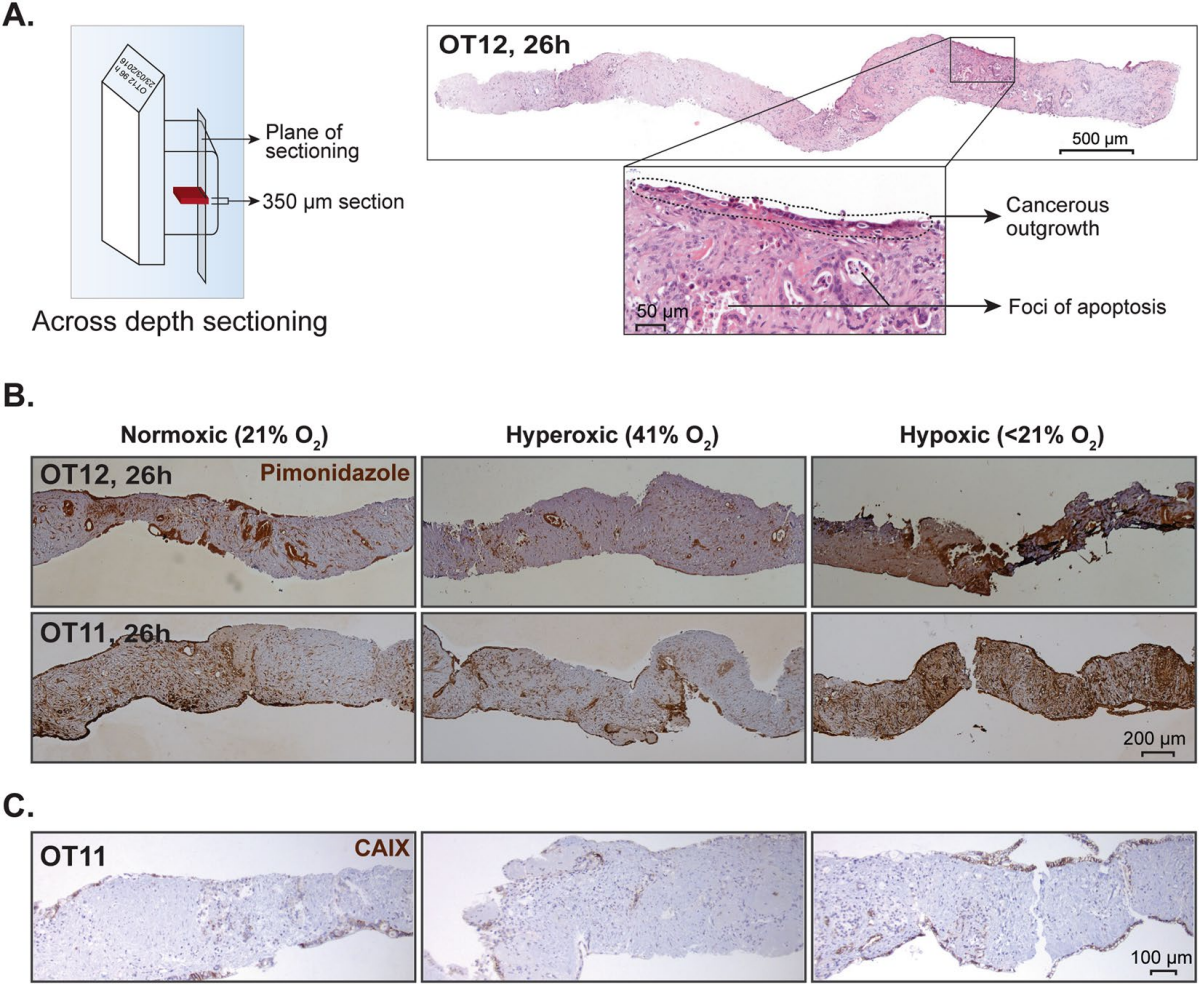
Schematic diagrams for analysis of tissue oxygenation. (**A**) Left panel, Vertical, i.e. across depth, tissue sectioning strategy. Right panel, A representative H&E-stained section of a vertically cut tissue slice. Inset demonstrates a higher magnification image with outgrowing cells and small foci of apoptosis. (**B**) Pimonidazole and (**C**) CAIX immunostaining of tissue slices cultured in normoxic (21% O_2_), hyperoxic (41% O_2_), and hypoxic (<21% O_2_) conditions.

Following successful preservation of tissue viability at an elevated oxygen level (hyperoxic, 41%), tissue oxygenation at ambient oxygen level (normoxic, 21%) was investigated. Tissue slices cultured in hypoxic condition (<21%) served as a positive control and exhibited extensive and strong pimonidazole staining as anticipated. Pimonidazole staining did not differ between normoxic and hyperoxic conditions. Ductal epithelial cells showed strong pimonidazole staining, even under hyperoxic condition, irrespective of the distance of the epithelial cells to the tissue surface facing the culture medium (Fig. 9A,B). Similar observations were made with CAIX staining, but this was found to be less sensitive than pimonidazole staining (Fig. 9C).

**Figure 10:**
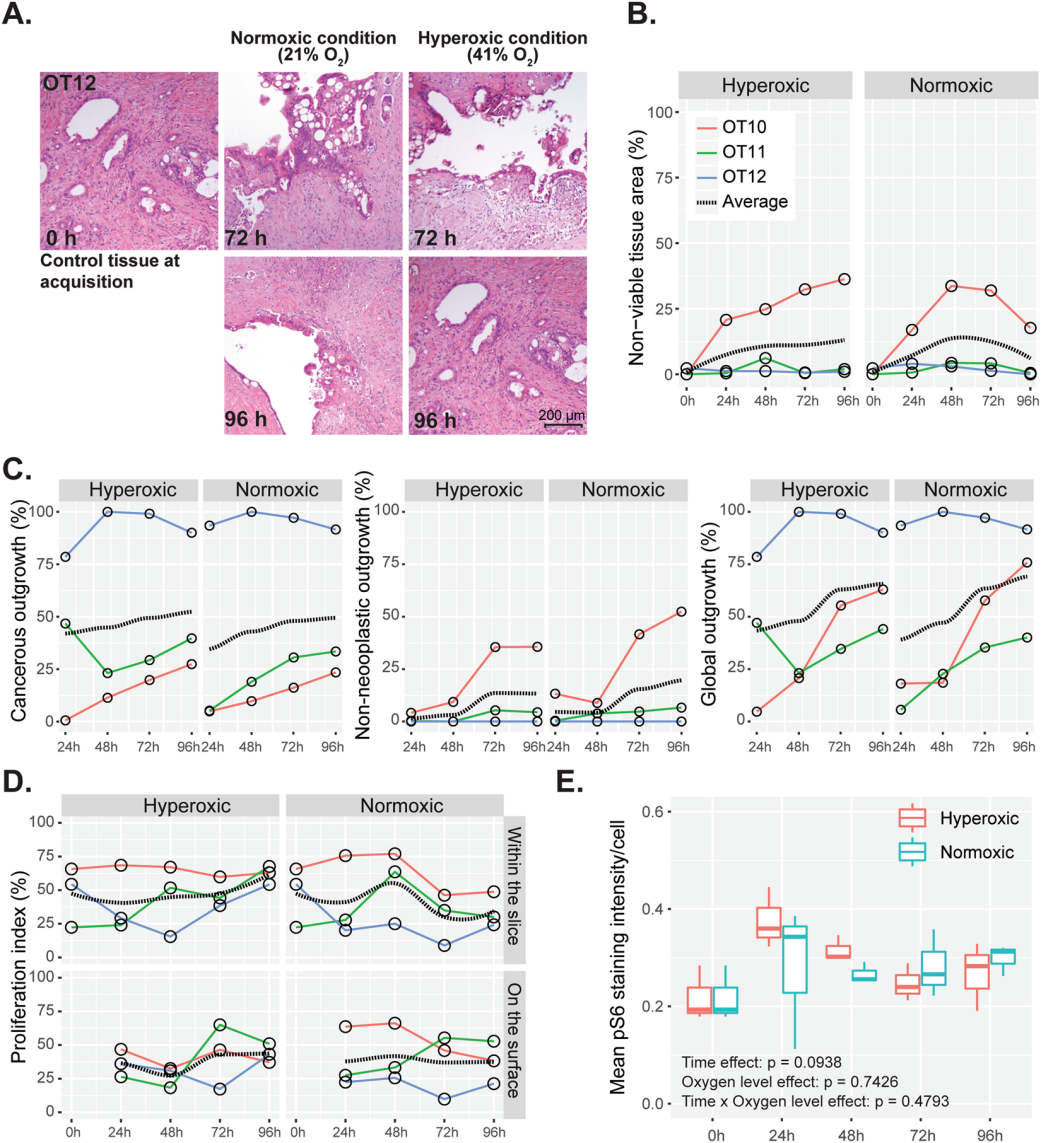
Tissue viability, cellular outgrowth, proliferation and metabolic activity at different oxygen levels. (**A**) Comparison of tumor histomorphology under normoxic (21% O_2_) and hyperoxic (41% O_2_) conditions at different time points (H&E staining). (**B**) Tissue viability in paired slices obtained from the same donor samples (n = 3) when cultured under normoxic and hyperoxic conditions. The dotted black line shows the conditional mean smooth curve to demonstrate the pattern of observation under the test conditions. Legend keys are the same for (**C**,**D**). (**C**) Cancerous, non-neoplastic and global cell outgrowths in donor-paired tissue slices (n = 3). (**D**) Proliferation index of cancerous cells within and on the surface of slices at different time points when donor-matched tissue slices were cultured under normoxic and hyperoxic conditions. (**E**) Metabolic activity of cancerous cells when cultured under different ambient oxygen levels. (Repeated measure two-way ANOVA followed by Dunn’s multiple comparison; p > 0.05, n = 3).

Morphological analysis revealed neither qualitative differences in histological phenotype (Fig. 10A) nor quantitative differences in tissue viability, outgrowth of cancerous or non-neoplastic cells, proliferative activity of the cancer cells, and pS6 immunohistochemical staining between paired tissue slices cultured under normoxic or hyperoxic conditions (Fig. 10B–E, Supplementary Fig. S7).

## Discussion

Preclinical *in vitro* and *in vivo* models to study pancreatic cancer have been available for many years (reviewed in^22^) with the aim to develop effective treatment in order to improve survival for this patient group with dismal prognosis. Cell cultures, genetically-engineered mouse models and patient-derived xenografts mimic poorly the key attributes of human PDAC *in vivo*. In this context, the presented *ex vivo* culture method of precision-cut tissue slices is unique in that it allows the preservation of pancreatic cancer tissue in its entirety, i.e. the tumor cell population and the native microenvironment with its complex composition of acellular and cellular constituents including immune cells. The developed methodology can reproducibly yield good overall tissue viability as well as maintenance of structural integrity, both at the light microscopic and ultrastructural level, during at least 96 h of culture. Importantly, the malignant cell populations retain their grade of differentiation, proliferative activity and morphological phenotypes. Furthermore, we observed a satisfactory preservation of the cancer-associated stroma, both morphologically and regarding the expression of stromal markers, and infiltrates of lymphocytes and macrophages. As the 3-dimensional architecture of the cancer and associated stroma is not disrupted in this *ex vivo* culture system, it is anticipated that the interactions between these compartments are preserved – a highly sought-after feature especially in the context of drug testing. PDAC is characterized by marked heterogeneity, which has prompted the proposal of several classification systems based on gene expression profiles^23^. Because molecular heterogeneity, both at the genomic and epigenomic level, is the main cause for divergence in results of cytotoxic and targeted treatments, the *ex vivo* system presented in this study has the significant advantage of allowing drug sensitivity testing of an individual patient’s pancreatic cancer. This model represents therefore a state-of-the-art tool in the development of precision medicine, as has been reported elsewhere with the explant culture of head and neck squamous cell carcinoma and colorectal cancer^24^. While patient-derived xenograft models offer in principle the same possibility of individualized treatment testing, these are more costly and time-consuming and have the important disadvantages of the tumor stroma being murine, not human, and the host immune system being absent. Furthermore, the proposed *ex vivo* culture system allows an in-depth characterization of the various subtypes of PDAC that are currently defined by gene expression analysis^25^ regarding cell biological functions and interactions with the tumor microenvironment. In addition to its main advantage of including the native tumor microenvironment, the presented culture system is relatively inexpensive, gives results within days, and helps reducing animal experiments.

Organotypic cultures have been established for various organs and tumors^10,26–28^, but when it comes to pancreas, efforts have mainly focused on normal pancreatic tissue^29^, and in particular the pancreatic stellate cells^12^, acinar cells^30^, and islets of Langerhans^31^. In a previous study, pancreatic cancer tissue slices were cultured as one of a panel of various cancer types, and it was shown that tissue slices retained some of the key features of the tumor *in vivo*, including cell proliferation and key signaling pathways^10^. Recently, it was suggested that the organotypic slice culture system may be used to investigate the immune microenvironment in PDAC^32^. In the present study, we have optimized the conditions for culturing of PDAC tissue slices during at least 96 h, and studied in depth the morphological integrity, immunohistochemical phenotype, and proliferative as well as metabolic activity of both the cancer and stromal cell populations to validate the adequacy of the system for future use in various experimental settings, including drug testing and optimization of the treatment of individual patients (precision medicine). Indeed, assessment of drug effects in tissue slices with compromised structural integrity or physiological functions may result in overestimation.

The pancreas, and in particular its acinar cell compartment, is known to be highly sensitive to ischemic conditions, both *in vivo*^33,34^ and *in vitro*^35^. To avoid tissue injury associated with warm ischemia, it is important to maintain low temperature during the entire tissue sampling and slicing procedure and to minimize transportation and processing time. Furthermore, tissue oxygenation needs to be uncompromised during culture. A slice thickness of 300–350 µm, as used in the current study, represents the maximum width at which diffusion of oxygen and substrates into the innermost cell layers of the tissue slice is still preserved. The oxygen concentration at the cell surface under 2.0 mm of 2D culture medium is between 10% and 50% of the concentration at the air-liquid interphase and further decreases as the total number of cells increases^36^. Assuming similar oxygen diffusion, our culture conditions ensured sufficient oxygen availability to the tissue slices in culture, as shown with pimonidazole staining of tissue slices cultured at 21% and 41% oxygen tension. Maintenance of comparable tissue viability at ambient (21%) oxygen tension suggests that tissue oxygenation was not a limiting factor. This is in line with previous investigations that indicated good tissue viability at normoxic condition for other tissues^10,37^. The use of an insert with a constant volume of medium below and above it (less than 1.0 mm depth above the slices) was adopted to prevent drying of the tissue slices and to maintain a relatively constant diffusion distance for tissue oxygenation, while a substantial medium volume beneath ensures efficient exchange of nutrients and metabolites (for technical details see Fig. 1). Inserts also facilitated the exchange of culture medium for experimental manipulation without the risk of inflicting mechanical damage to the tissue slices. Culturing of submerged tissue slices is likely to be particularly useful for drug testing or any experimental manipulation requiring unrestricted access of soluble molecules to the tissues.

During culture, an increasingly extensive cellular outgrowth along the surface of the tissue slices was consistently observed. It was composed of either pancreatic cancer or non-neoplastic, normal-appearing ductal epithelial cells, the latter apparently originating from transected ducts at the cut tissue edge. The outgrowth of duct epithelial cells showed signs of progressive maturation during culture, as indicated by the gradual transition from a flattened cell morphology during the first days to a high, cylindrical one at the termination of culture. This phenomenon, reminiscent of “wound healing”, may warrant further investigation in the context of tissue regeneration. The cancerous cell outgrowth characterized by cytological and architectural atypia, retained the degree of differentiation seen in the part of the tumor growing inside the tissue slice. Outgrowth onto the slice surface was prominent in well and moderately differentiated carcinomas but limited in poorly differentiated cancers. The reason for the difference in cancer cell outgrowth is unknown but may be linked to divergence in cancer cell motility and interaction with the stroma. This phenomenon deserves further investigation and careful observation in future experiments on PDAC, including drug testing, based on precision-cut tissue culture.

An important finding in our study is the fact that in the presented culture system, tissue damage amounting to apoptosis or necrosis was overall not significantly increased compared to control tissue, and represented on average no more than 5% of the total tissue area. However, mapping of the foci of apoptosis and necrosis revealed that the peripheral and intermediate regions of the tissue slices were more affected than the central zone. This observation together with the early occurrence of these changes (within the first 24 h) point at a possible mechanical rather than a hypoxia-related cause for the tissue damage. Of further relevance is the finding that there were no appreciable changes in the morphology of the tumor microenvironment and that analysis of the marker expression profile of the main constituting cell populations (cancer-associated fibroblasts, lymphocytes and macrophages) confirmed preservation of the cellular heterogeneity that is characteristic for the non-neoplastic tumor compartment. Hence, cultures of precision-cut PDAC slices may be an unparalleled model to analyze the complex interactions between the cancer cell population and the stroma, which according to recent results affect nearly all aspects of tumor biology and are considered for stroma-specific treatment strategies of PDAC^38^.

Of further importance is the observation that overall, the proliferative activity of the cancer cells remained stable during culture and, in particular, that there was no significant change compared with the non-cultured control tissue. However, as results varied between individual cases, assessment of the proliferative activity in source tissue and cultured tumor slices will probably have to be an integral part of the procedure when using this system for drug testing purposes, because culture-induced changes in proliferation – as they have been described in explant cultures of other cancer types^39^ - may require a shortened time of drug exposure. Importantly, oxygen tension did not seem to affect the proliferative activity of the tumor. Our observations also indicate that the difference in proliferative activity between individual cases reflected the degree of tumor differentiation, which is relevant with a view of using this culture system for personalized drug testing. However, as the number of tumors with various grades of differentiation was small in this study, further investigation of this important aspect in a larger series is needed.

Metabolic (mTOR) activity, an essential feature of (cancer) cell biology that is particularly relevant with a view to drug sensitivity testing, was found to be increased in the tissue slices when cultured *ex vivo*, as has been reported by others^10,39^. Higher metabolic activity in tissue slices at 24 h is not surprising given the exposure to stress related to the slicing process and the subsequent adaptive response characterized by cellular outgrowth onto the tissue surface, processes which are associated with increased energy use. Placing the freshly cut slices in nutrient-rich medium further provides conditions that fuel metabolic activity. The regulation of mTOR activity in relation to external stimuli merits mentioning here as a possible explanation. Likely most relevant in the context of *ex vivo* culturing is the nutrient-sensing function of mTORC1^40^: increased availability of nutrients, mainly glucose and amino acids, augments the activity of mTORC1, which in its turn results in increased pS6 levels. *Ex vivo* culture media often contain these nutrients at higher levels than those found in human plasma, which potentially could augment the activity of mTORC1. Further studies are warranted to determine the mechanistic basis of augmented metabolic activity in precision-cut tumor slices when cultured *ex vivo*. Increased metabolic activity in *ex vivo* culturing requires also careful consideration when using the culture system for drug testing, both regarding the predictive power and the optimal timing of drug exposure.

The culture system described in this study has certain limitations. Although well suited for precision medicine, it is not currently adapted for high-throughput analysis or long-term (weeks to months) culture. Lack of circulation of both blood and lymph limits the possibility of migration of inflammatory/immune cells into the tissue. Non-physiological drainage of the pancreatic secretome is likely to have an effect that is mainly limited to residual acini scattered between tumor cell clusters. Diffusion of drugs into the central parts of the tissue slice may be restricted, especially for larger compounds. A further important limitation of the culture model results from the highly dispersed growth that is characteristic of PDAC and leads inevitably to inter-slice variability in terms of both tissue composition and tumor cell content in precision-cut slices. This may be an obstacle especially for the evaluation of drugs that have a moderate effect and therefore require precise quantification. Furthermore, given that intratumor heterogeneity is common and may affect key tumor features, some of which were investigated in this study, e.g. grade of differentiation, proliferation and metabolic activity, testing of multiple cancer tissue slices that are sampled from different tumor areas may be required^41^. As neoadjuvant treatment is increasingly becoming part of the management of patients with PDAC, there is a clinical need for the prediction of the therapeutic effect based on biopsy material. However, the highly limited amount of tissue contained in these samples represents a challenge that requires specific testing. All of these limitations should be considered when using the system for drug testing. To this end, our next aim is to test the presented culture system for drug sensitivity screening.

## Supplementary information

Supplementary Data

## Data Availability

Request for additional data presented in this manuscript shall be forwarded to the corresponding author.
