# Supplementary material for: Ex vivo organotypic culture system of precision-cut slices of human pancreatic ductal adenocarcinoma
Source: Sci Rep. 2019 Feb 14;9:2133. doi: 10.1038/s41598-019-38603-w (PMC6376017; doi:10.1038/s41598-019-38603-w)
Supplement: Supplementary file 1 — Supplementary Data [file 41598_2019_38603_MOESM1_ESM.pdf]

Supplementary Information

Ex vivo organotypic culture system of precision-cut slices of human pancreatic ductal adenocarcinoma

Sougat Misra[1, §], Carlos F. Moro[1,3 §], Marco Del Chiaro[2], Soledad Pouso[3], Anna Sebestyén[4], Matthias Löhr[2], Mikael Björnstedt[1], Caroline S. Verbeke[3,5,6]

[1]*Department of Laboratory Medicine, Division of Pathology F46, Karolinska Institutet, Karolinska University Hospital Huddinge, SE-141 86 Stockholm, Sweden*

[2]*Department of Clinical Intervention and Technology (CLINTEC), Center for Digestive Diseases, Karolinska University Hospital and Division of Surgery, Karolinska Institutet, Stockholm 14186, Sweden*

[3]*Department of Clinical Pathology/Cytology, Karolinska University Hospital, Stockholm SE-141 86, Sweden*

[4]*Tumour Biology Laboratory, 1st Department of Pathology and Experimental Cancer Research, Semmelweis University, Budapest 1085 Ulloi ut 26., Hungary*

[5]*Institute of Clinical Medicine, University of Oslo, Postbox 1171 Blindern, Oslo 0318, Norway*

[6]*Department of Pathology, Oslo University Hospital, Rikshospitalet, Postbox 4956 Nydalen, Oslo 0424, Norway*

Running title: Pancreatic cancer in a dish

***Corresponding author**: Caroline S. Verbeke

Department of Pathology, Institute of Clinical Medicine, University of Oslo, Oslo, Norway

E-mail: c.s.verbeke@medisin.uio.no, caroline.verbeke@ki.se

Tel. +47-23071498, Fax. +47-23070001

Key words: Pancreas, adenocarcinoma, ex vivo, organotypic model, tissue slice technology

[§]Both authors have contributed equally

Supplementary Figure S1

Outline of the investigations

Preliminary basic optimization steps (data not shown)

Variables	Entities		
**1. Culture medium (n=3)**	DMEM + 10% FCS	*versus*	CMRL1066 + 2.5% human serum AB ☑
		↓ Use CMRL1066 + 2.5% human serum AB	
**2. Antioxidant supplement (n=3)**	Pycnogenol and/or N-acetyl cysteine ☑	*versus*	No supplementation
		↓ No supplementation with antioxidants	
**3. Tissue culture insert (n=3)**	Use of insert ☑ (Millicell®, Millipore)	*versus*	Free-floating tissue slices in culture
		↓ Use insert	
**4. DPDS supplement (n=2)**	100 nmol/L ☑	*versus*	No supplementation
		↓ Use DPDS	
		**Culture conditions defined**	

☑ (green) indicates entities the use of which resulted in better tissue survival
☑ (grey) indicates entities the use of which did not make any difference in tissue survival

Characterization of tissue slices (Fig. 2-8)

1. Tissue viability: H&E staining, TEM, quantification, n=12
2. Cell outgrowth: H&E staining, IHC, quantification, n=12
3. Proliferative activity: IHC, n=7
4. Immunohistochemical phenotyping:
    - cancer: n=2-6
    - microenvironment: n=2-6
5. Metabolic activity: quantification, n=5

Assessment of effect of oxygen tension (Fig. 9-10)

1. Hyperoxic (41%) versus normoxic (21%) conditions: n=3
2. Hypoxia assessment *in situ*: n=1-3
3. Histomorphological characterization: n=3
4. Tissue viability: H&E staining, quantification, n=3
5. Cell outgrowth: H&E staining, IHC, quantification, n=3

Supplementary Figure S1. A flow chart showing experimental progression and different endpoint analyses.

Supplementary Figure S2

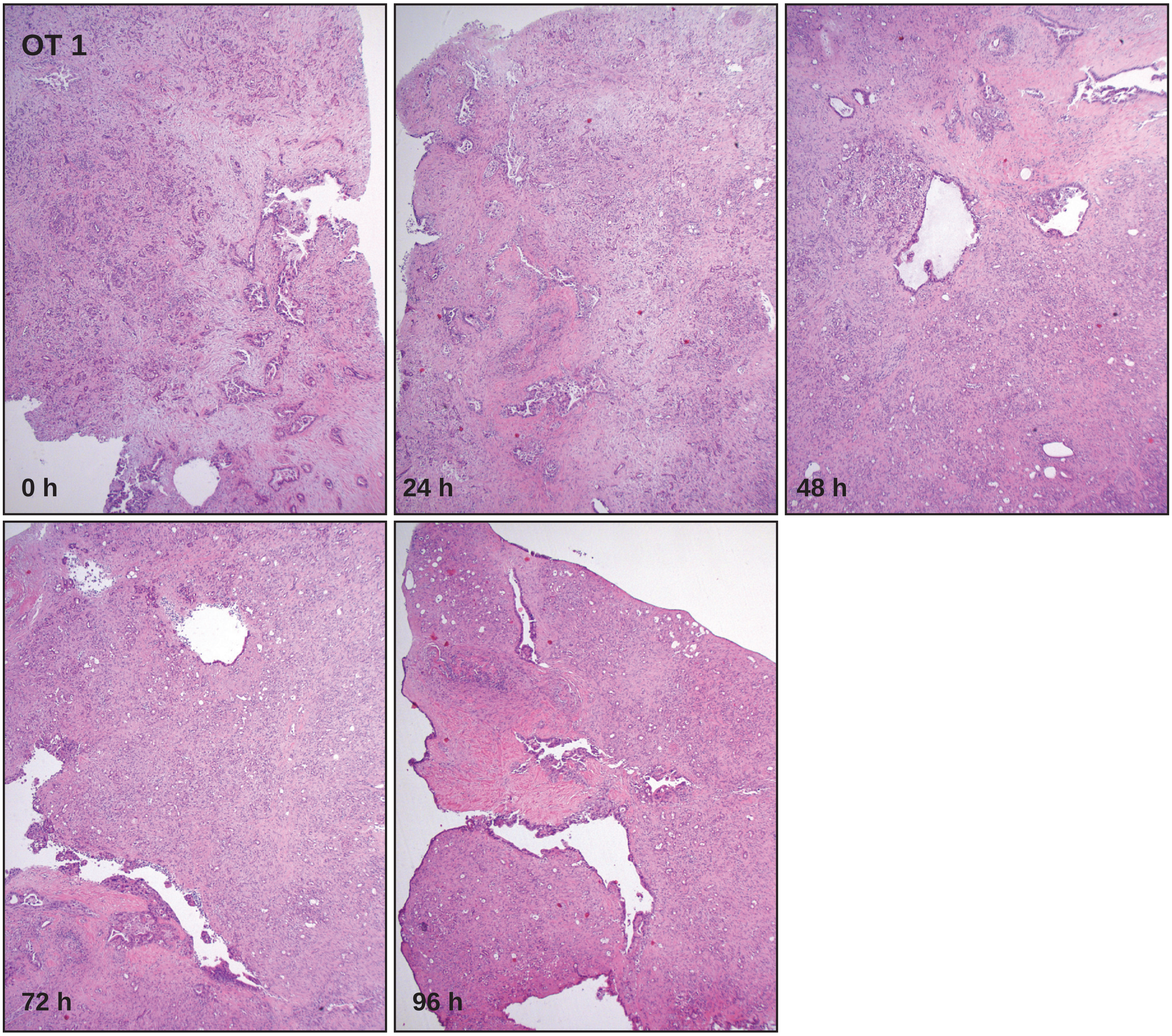

Supplementary Figure S2. Low magnification (4X) images of H&E-stained control (0 h) and cultured (24h -96 h) tissue slices.

Supplementary Figure S3

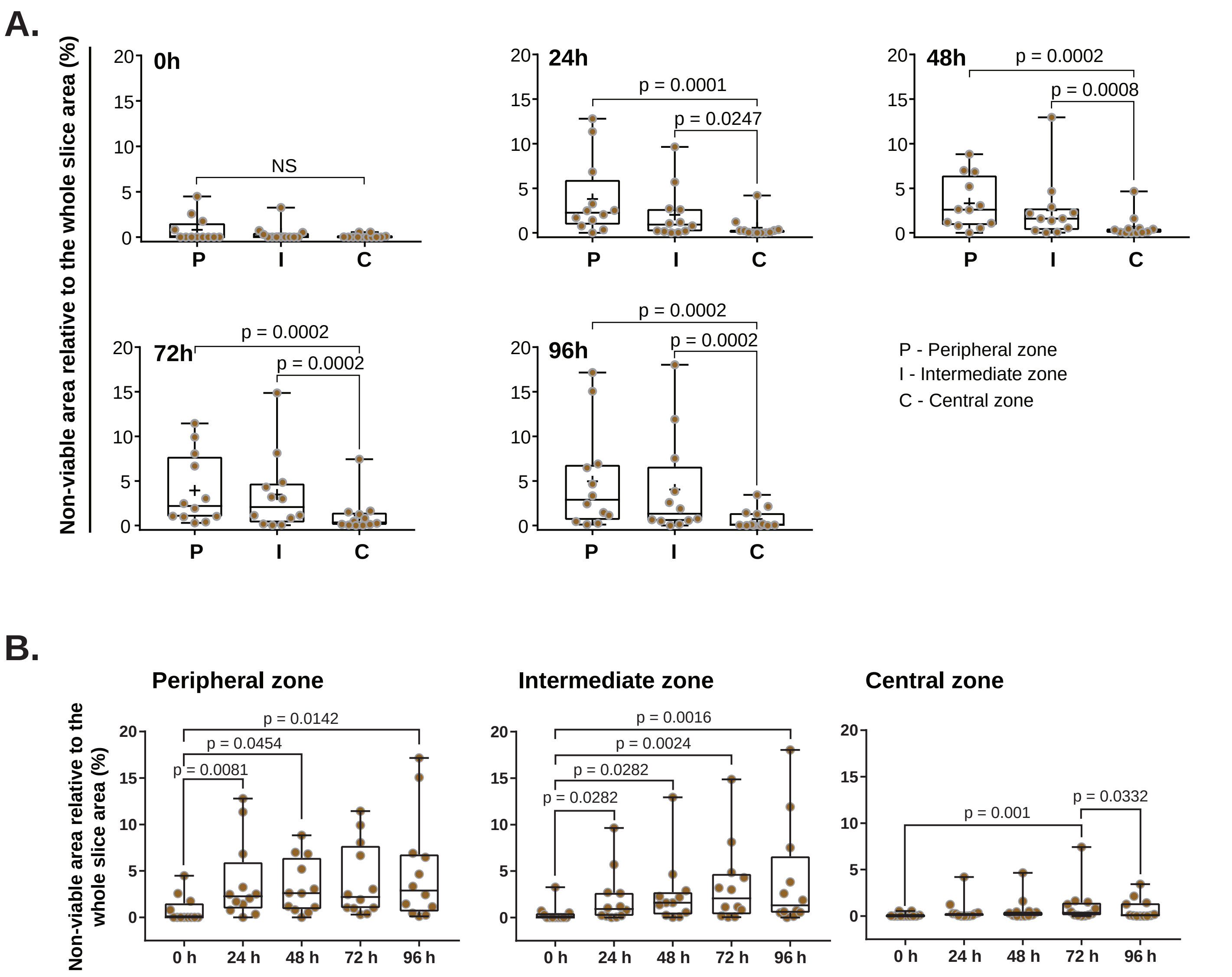

Supplementary Figure S3. (**A**) Quantification of non-viable tissue areas at different time points and for the three tissue zones. At culture initiation (0 h), there were no significant differences in the extent of non-viable tissue areas ($p > 0.05$). At later time points (24 h – 96 h), non-viable tissue area was significantly higher in the periphery and the intermediate zones than in the central zone (n=12; Friedman test followed by two-stage linear step-up multiple comparison procedure according to Benjamini, Krieger and Yekutieli; $p < 0.05$). (**B**) Temporal changes in tissue viability within a defined zone. In all zones, significant changes in tissue viability were observed compared to the control at 0 h (similar statistical analysis as above; $p < 0.05$). Following damage within the first 24 h of culture, no further increase in non-viable tissue area could be observed, except for the central zone in which tissue viability was significantly lower at 72 h.

Supplementary Figure S4

A.

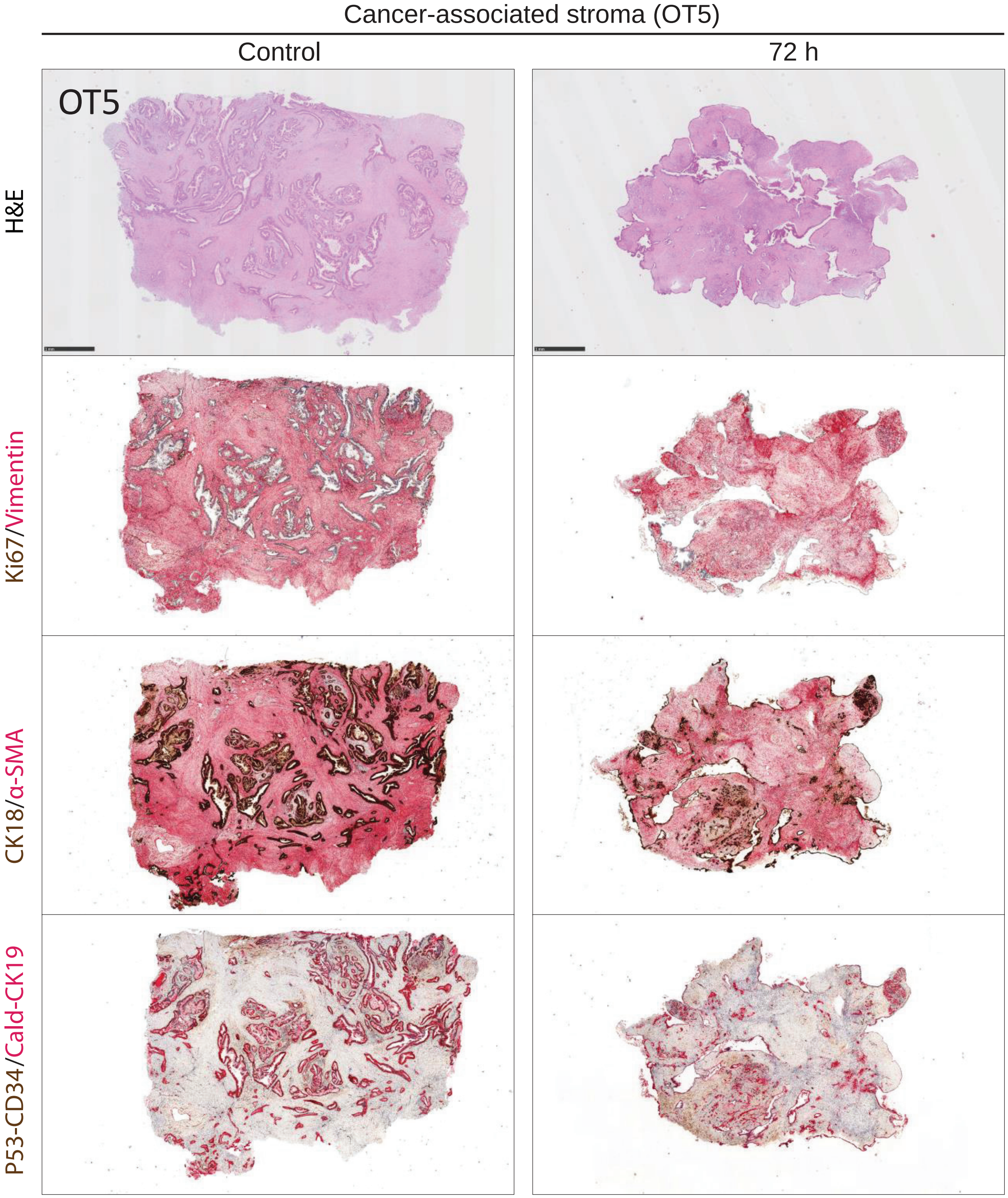

continued...

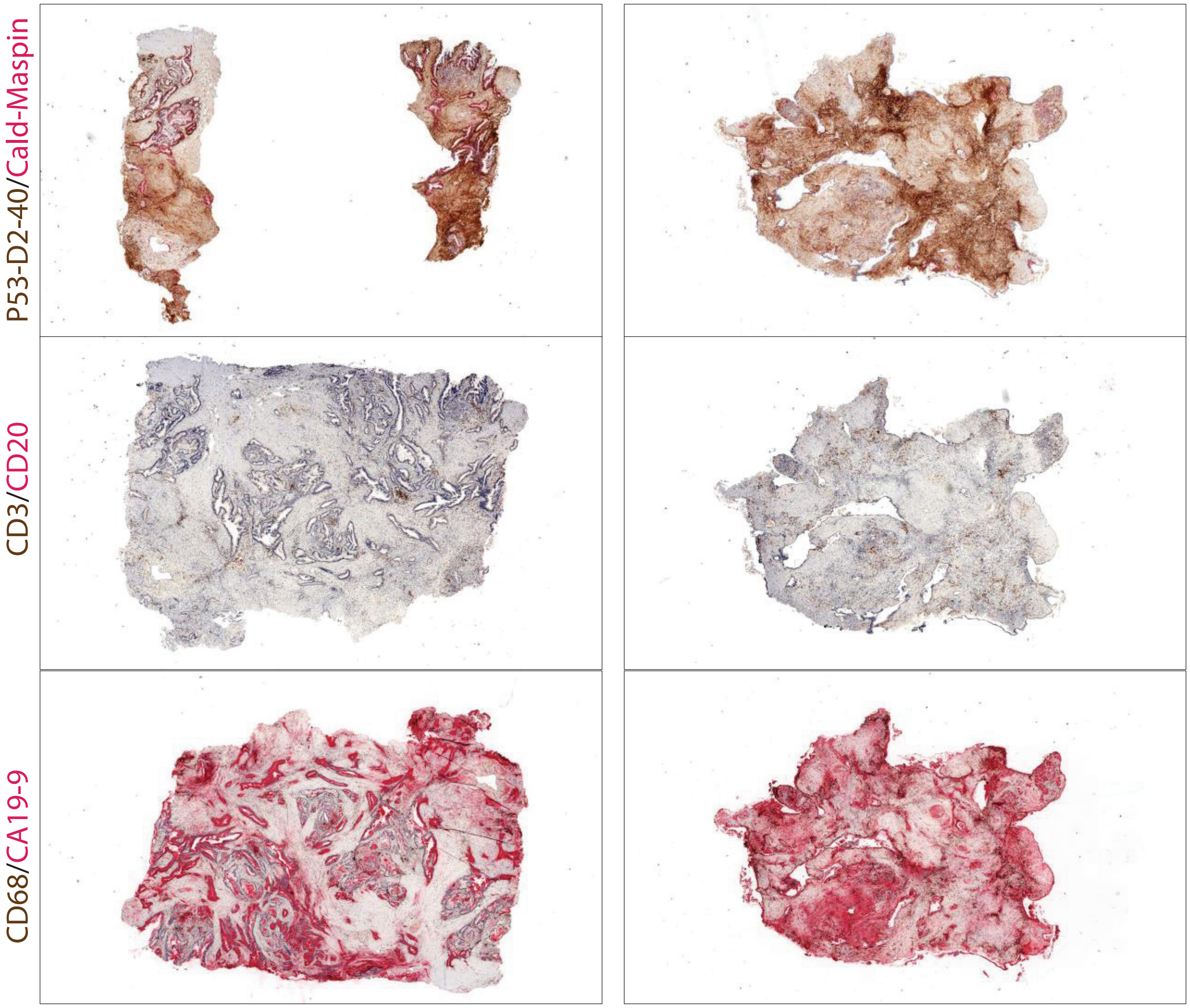
P53-D2-40/Cald-Maspin
CD3/CD20
CD68/CA19-9

B.

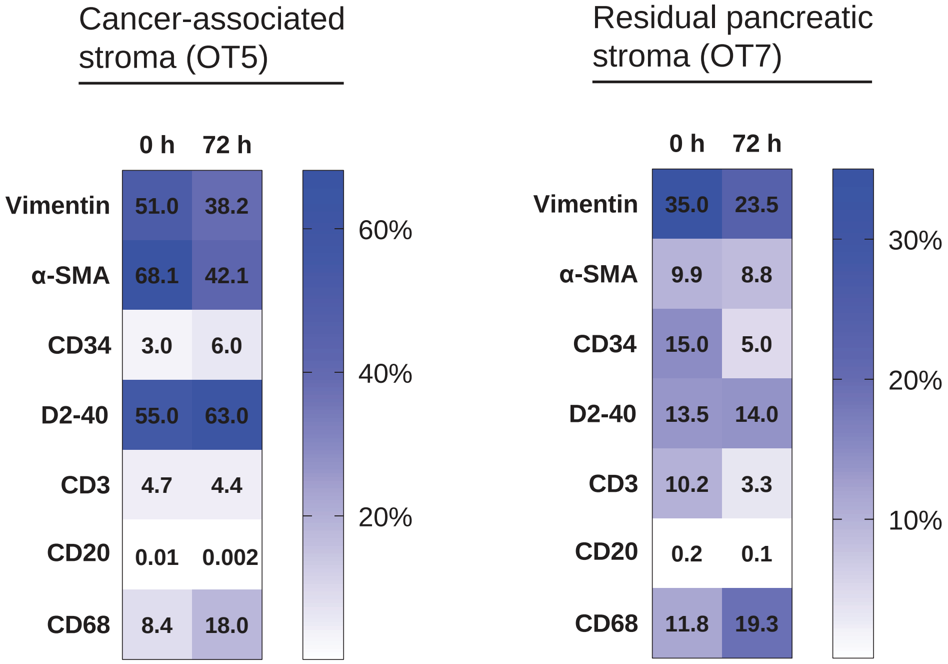
Cancer-associated stroma (OT5)
0 h
72 h
Vimentin 51.0 38.2
α-SMA 68.1 42.1
CD34 3.0 6.0
D2-40 55.0 63.0
CD3 4.7 4.4
CD20 0.01 0.002
CD68 8.4 18.0
60%
40%
20%
Residual pancreatic stroma (OT7)
0 h
72 h
Vimentin 35.0 23.5
α-SMA 9.9 8.8
CD34 15.0 5.0
D2-40 13.5 14.0
CD3 10.2 3.3
CD20 0.2 0.1
CD68 11.8 19.3
30%
20%
10%

Supplementary Figure S4. (**A**) Photomicrographs of control (0 h) and cultured (72 h) organotypic slices from a well differentiated tumor (OT5) stained with immunohistochemical markers for the characterization of stroma (vimentin, αSMA, CD34, D2-40) and immune cells (CD3, CD20 and CD68). (**B**) Quantification of stroma and immune markers, expressed as their extent (%) in the whole tissue area.

Supplementary Figure S5

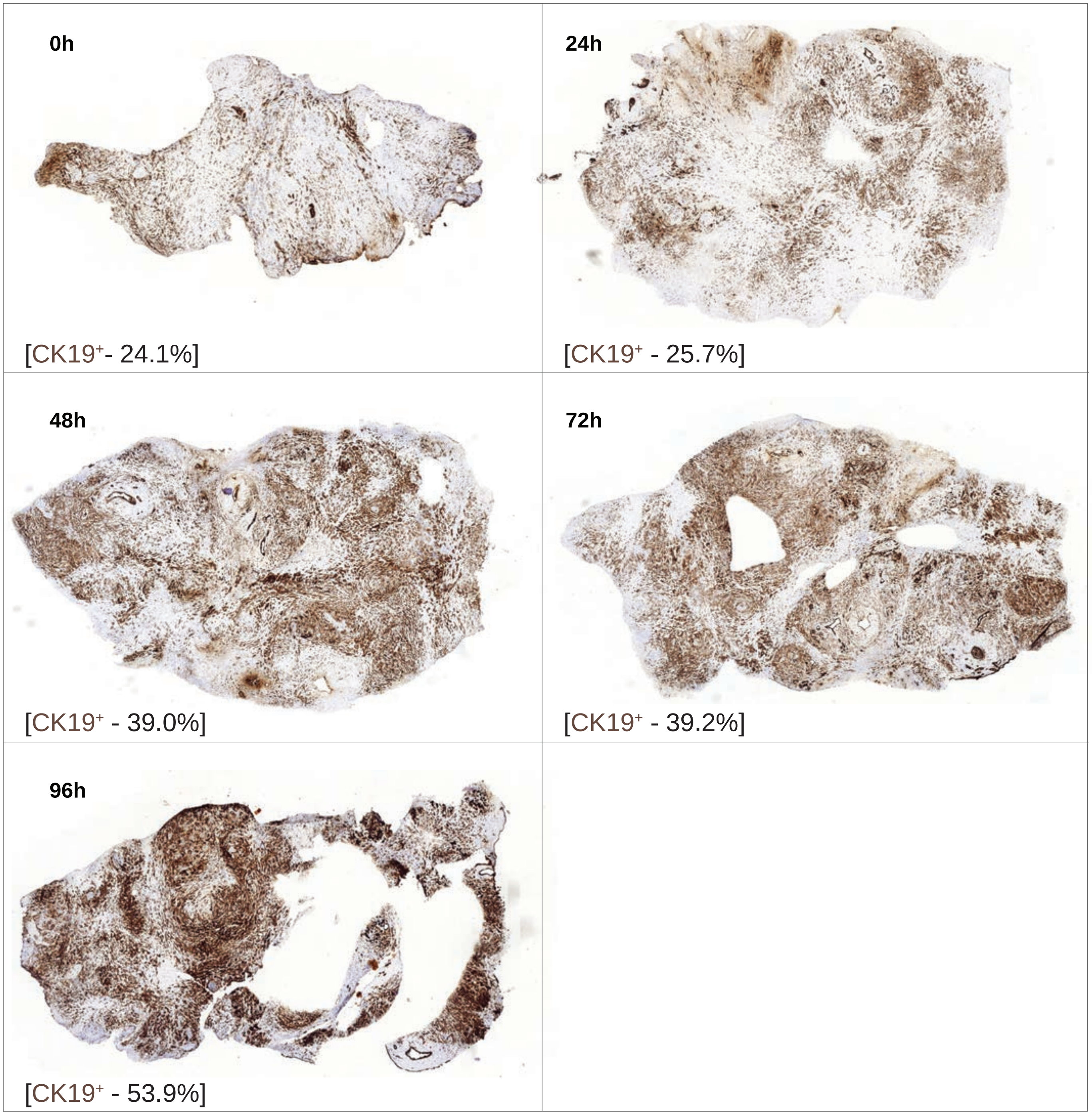

Supplementary Figure S5. Immunohistochemical staining for CK19 (OT2) at all time points, showing a progressive increase in the number of cancer cells within the tissue slice. CK19 positivity indicates area occupancy of cancerous cells within the tissue slices.

Supplementary Figure S6

A.

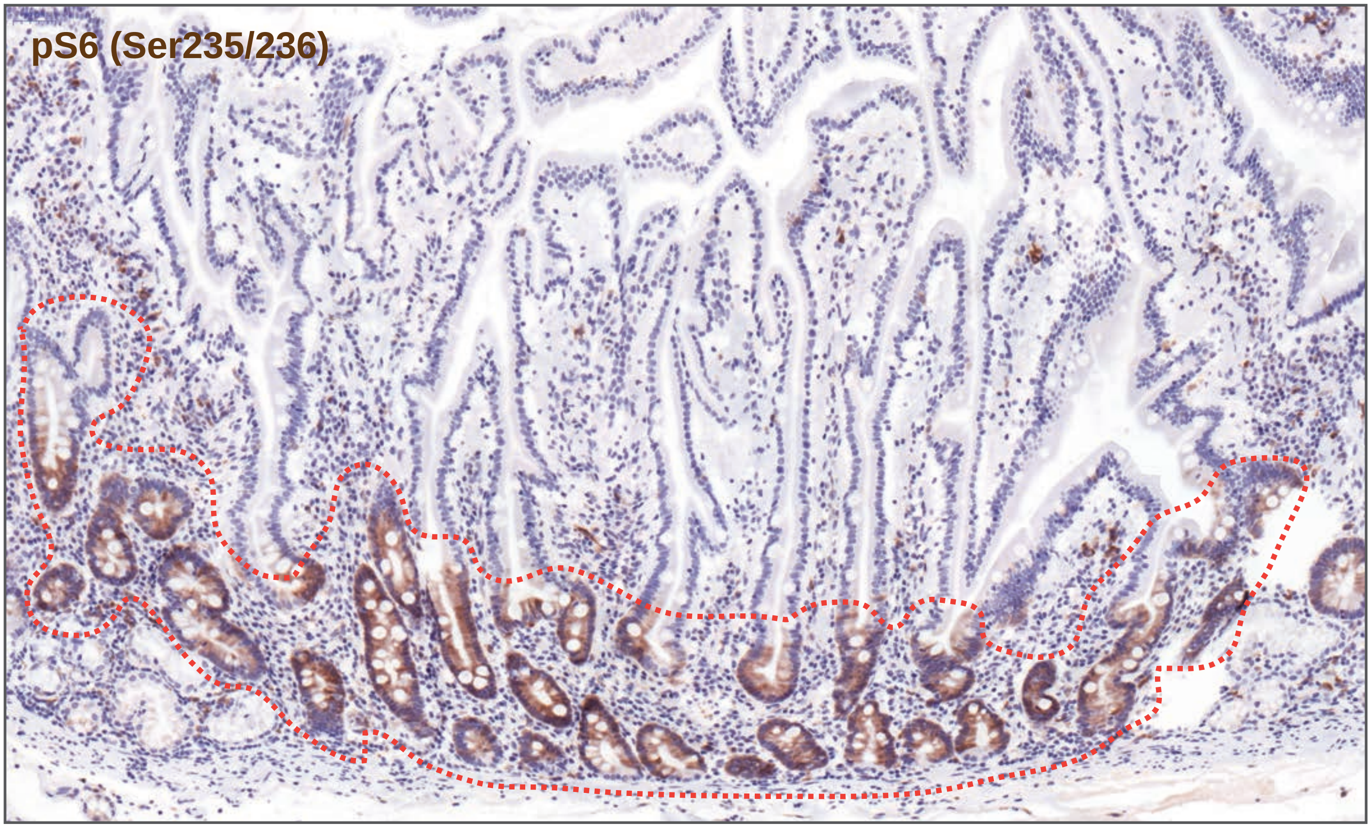

B.

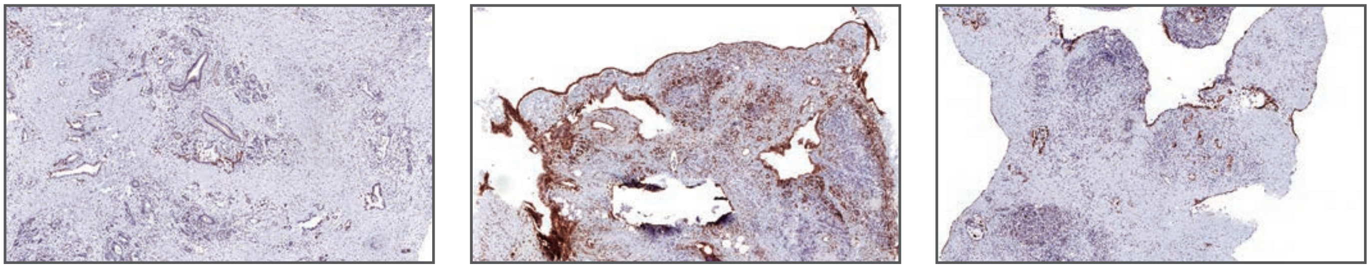

Control tissue at acquisition   Control tissue after 96 h culture   Rapamycin treatment - 72 h

Supplementary Figure S6. (**A**) Positive immunohistochemical staining for pS6 in duodenal crypt cells was used as a positive control. (**B**) pS6 staining in cultured tissue slice either untreated or subjected to rapamycin (50 nM) treatment for 72 h. Matched control tissue at acquisition was used to assess the pS6 positivity prior to culture.

Supplementary Figure S7

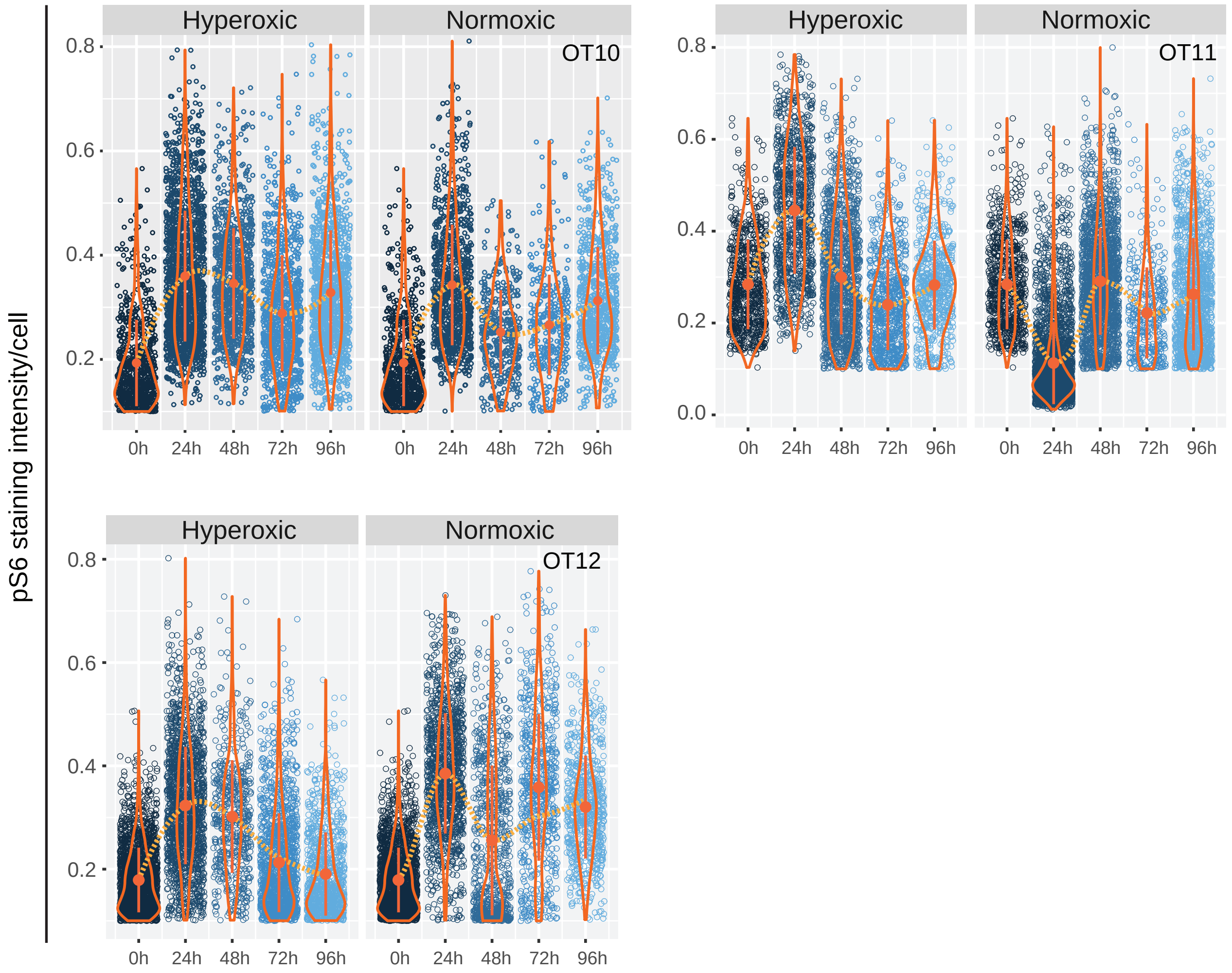

Supplementary Figure S7. Temporal changes in pS6 staining intensity of cancerous cells in patient-matched tissue slices under hyperoxic or normoxic conditions (n=3). The dotted lines show the conditional mean smooth curve fitted to the data points.

Supplementary Table 1: Antibody list for immunohistochemical analysis.

**Antibody**	**Clone name**	**Dilution**	**Product code**
Actin (smooth muscle)	1A4	1:500	M0851
CA19-9	CA241:5:1:4	1:400	NCL-L-CA19-9
CAIX	TH22	1:25	NCL-L-CAIX
Caldesmon	h-CD	1:300	M3557
CD20	L26	1:1000	M0755
CD3	LN10	1:100	NCL-L-CD3-565
CD34	QBEnd/10	1:50	M7165
CD68	PG-M1	1:100	M0876
CK18	DC-10	1:50	M7010
CK19	b170	1:100	NCL-CK19
D2-40	D2-40	1:50	M3619
Ki-67	MIB-1	1:100	M7240
Maspin	G167-70	1:300	BDP 554292
p53	DO-7	1:300	NCL-L-p53-DO7
SMAD4	B-8	1:300	sc-7966
pS6 ribosomal protein		1:100	2211S
Trypsin	Not available	1:100000	MAB1482
Vimentin	V9	1:1500	M0725

Antibody suppliers:

BDP: BD Pharmingen - BD Biosciences, Franklin Lakes, New Jersey, United States.
M: Dako - Agilent: Dako, Glostrup, Denmark.
MAB: Millipore – Merck, Billerica, Massachusetts, United States.
NCL: Novocastra Leica Biosystems Ltd, Newcastle Upon Tyne, United Kingdom.
sc: Santa Cruz Biotechnology, Inc., Dallas, United States.
pS6 – Cell Signaling Technology, Leiden, Netherlands.

All listed antibodies were stained using a Leica BOND III automated immunostainer, with exception of CAIX and pS6 that were stained in a Ventana Benchmark Ultra immunostainer.
